# Evolution of Long‐Term Social Vulnerability After Tropical Cyclones in the United States

**DOI:** 10.1029/2025GH001727

**Published:** 2026-07-24

**Authors:** Lingke Jiang, Robbie M. Parks, Yoshira Ornelas Van Horne, Xiao Wu

**Affiliations:** ^1^ Columbia University Mailman School of Public Health Department of Biostatistics New York NY USA; ^2^ Columbia University Mailman School of Public Health Department of Environmental Health Sciences New York NY USA; ^3^ UCLA Fielding School of Public Health Department of Health Sciences Los Angeles CA USA

**Keywords:** social vulnerability, synthetic control methods, tropical cyclones, post‐disaster gentrification, internal migration

## Abstract

Tropical cyclones pose a substantial threat to the health and welfare of communities across the United States. While existing research has focused primarily on the short‐term impacts of tropical cyclones, typically within days or months after exposure, there remains a gap in understanding their long‐term effects on the socioeconomic and demographic composition of communities–a crucial factor in measuring community resilience and recovery. In this study, we conducted a synthetic control analysis using data from all tropical cyclones that occurred in the United States during 2005–2018. We provide evidence that social vulnerability initially increased after tropical cyclones, but exposed regions recovered over time and became less socially vulnerable in the long term. For exposed regions, the social vulnerability index (SVI) increased by 2.4% (95% confidence interval (CI), 0.7%–4.1%) in the year after tropical cyclones, and persisted at elevated levels for at least 5 years. However, SVI decreased over time and, 12 years after exposure, fell even lower than expected in the absence of a tropical cyclone, decreasing to −1.0% (95% CI, −1.9%–0.0%). Our study suggests that this long‐term trend may reflect post‐cyclone gentrification, where vulnerable communities are displaced and replaced by more affluent, less vulnerable populations.

## Plain Language Summary

Tropical cyclones remain a persistent threat across the United States, with profound social impacts on individuals and communities. This study empirically quantifies the multiyear changes in social vulnerability following tropical cyclones, considering all tropical cyclone events during 2005–2018. Our findings reveal a spike in social vulnerability in the year after tropical cyclones, but over time, affected regions tend to recover and experience post‐disaster gentrification, leaning toward lower social vulnerability compared to similar regions not affected by tropical cyclones in the long term. This trend may be attributed to resource allocation in disaster response along with post‐disaster displacement and economic migration.

## Key Points

- We conducted a synthetic control analysis using data from all tropical cyclones that occurred in the contiguous US during 2005–2018
- Social vulnerability initially spiked post‐exposure, but exposed regions recovered over time and became less vulnerable in the long term
- This long‐term trend points to post‐disaster gentrification

## 1 Introduction

Hurricanes and tropical storms, collectively known as tropical cyclones, are fast‐spinning storm systems with a low‐pressure center that produce strong winds and spiraling thunderstorms (Anthes, 2016) and can cause extensive infrastructure damage. From 1980 to 2024, the costs related to tropical cyclones in the United States (US) are estimated to have exceeded $1.3 trillion (NOAA National Centers for Environmental Information, 2024). The risk of tropical cyclones for nearly 127 million people living in coastal communities in the US is further aggravated by climate change, which has been shown to increase hurricane frequency for the Gulf and Lower East Coast regions (Balaguru et al., 2023). The immediate effects of tropical cyclones on individual health are widely documented and have been associated with physical injuries, mental stress, and increased risk of diseases (Huang et al., 2024; Parks et al., 2022, 2023; Shultz et al., 2005). A 2022 study revealed that death rates increased in the month following tropical cyclones for several causes, including cardiovascular, neuropsychiatric, and respiratory diseases (Parks et al., 2022). A following large‐scale mortality evaluation for all tropical cyclones in the contiguous US during 1930 and 2015 found a robust increase in excess mortality that persists for up to 15 years (Young & Hsiang, 2024). Housing conditions may worsen after tropical cyclones, and access to healthcare services and medication supplies may experience severe disruptions (Shultz et al., 2018). The existing literature has documented economic impacts based on data from community‐level income and employment (Coffman & Noy, 2012; Groen et al., 2020; Yabe et al., 2020). Collectively, tropical cyclones create devastating and wide‐ranging impacts on society, affecting both individuals and communities.

The impacts of tropical cyclone exposures vary greatly between communities, revealing underlying inequality (Parks et al., 2022). Historically marginalized communities not only suffer greater impacts after tropical cyclones, but also receive unequal benefits from recovery programs (Burrows et al., 2023; Reid, 2013). In the US, communities with predominantly black or low‐income populations have historically faced greater challenges in securing federal aid and housing insurance following disasters (Greenberg, 2014). Post‐disaster recovery efforts following major hurricanes often deepen urban inequality through a process known as “crisis‐driven urbanization” (Greenberg, 2014). Following Hurricanes Katrina (2005) and Sandy (2012), narrative evidence shows that redevelopment in affected areas tends to be highly uneven, with fortified wealthy neighborhoods, catalyzed gentrification and displacement in low‐income areas, and a pattern where disasters become opportunities for powerful coalitions to pursue redevelopment projects (Aune et al., 2020; Van Holm & Wyczalkowski, 2019). Furthermore, the housing crisis in New Orleans after Katrina saw massive displacement and a severe shortage of rental unit replacement (Comerio, 2014). Paradoxically, this inequitable rebuilding may be fueled by the very aid designed to mitigate disaster impact. While most storms depress local economies, the largest hurricanes often don't show the same negative impact on income, likely because they trigger massive federal aid packages that, while stabilizing the economy, can also finance this uneven redevelopment (Callahan et al., 2024).

Understanding the long‐term effects of tropical cyclone exposures on the dynamics of social vulnerability in affected communities is crucial to disaster management, public health, and social welfare. However, our knowledge of how socioeconomic and demographic compositions evolve in these affected communities over time remains limited (Ghosh et al., 2022), for two main reasons. First, previous studies have focused mainly on the relatively short‐term impacts of tropical cyclones (Bell et al., 2023; Ghosh et al., 2022, 2022; Lawrence et al., 2019; Parks et al., 2021, 2022, 2023; Weinberger et al., 2024). Second, a lack of harmonized longitudinal data and suitable analytical methods has limited most studies to assessing single tropical cyclone events rather than the cumulative effects of multiple disasters (Coffman & Noy, 2012; Groen et al., 2020; Karbownik & Wray, 2019).

Here, we empirically quantify the impacts of all tropical cyclone exposures that occurred during 2005–2018 in the contiguous US on the following multi‐year changes of social vulnerability in affected regions, as measured by the Centers for Disease Control and Prevention (CDC) social vulnerability index (SVI), a comprehensive metric derived from numerous socioeconomic and demographic factors to characterize community social vulnerability at county‐level (B. E. Flanagan et al., 2011). Collecting multi‐year tropical cyclones, meteorological, socioeconomic, and demographic data and applying an extended synthetic control approach, we assessed how social vulnerability in counties affected by tropical cyclones would have changed if they had not been exposed to tropical cyclones and then compared these counterfactual changes with their actual evolution. To validate the significance of the effects, we conducted placebo tests to rule out the possibility that the observed effect could have occurred by chance alone (Abadie et al., 2010). We made all data and statistical modeling procedures publicly available.

## 2 Materials and Methods

### 2.1 Data

Social vulnerability refers to the potential susceptibility of communities to negative effects caused by external stresses on human health, which helps to identify communities that need support before, during, or after disasters. The SVI provided by the Centers for Disease Control and Prevention/Agency for Toxic Substances and Disease Registry (CDC/ATSDR) is a comprehensive measure of community social vulnerability based on numerous US census variables from both the decennial US Census and the 5‐year American Community Survey (ACS), as detailed in Table 1 (Centers for Disease Control and Prevention Agency for Toxic Substances Disease Registry, 2025). These variables were categorized into four themes: Socioeconomic Status, Household Characteristics, Racial and Ethnic Minority Status, Housing Type and Transportation. The overall SVI is an additive index constructed by summing the percentile ranking of these four themes, which were then ranked against all other counties to generate the final percentile ranking. SVI is a percentile ranking that ranges from 0 to 1, with values closer to 0 indicating lower vulnerability and those closer to 1 indicating higher vulnerability. The methods to construct the SVI have been detailed in the literature (B. E. Flanagan et al., 2011).

**Table 1:** Compositions of Current Overall Social Vulnerability Index

Socioeconomic status	Below 150% poverty
	Unemployed
	Housing cost burden
	No high school diploma
	No health insurance
Household characteristics	Aged 65 years and older
	Aged 17 years and younger
	Civilian with a disability
	Single‐parent households
	English language proficiency
Racial and ethnic minority status	Hispanic or Latino (of any race)
	Black or African American, not Hispanic or Latino
	Asian, not Hispanic or Latino
	American Indian or Alaska Native, not Hispanic or Latino
	Native Hawaiian or Pacific Islander, not Hispanic or Latino
	Two or more races, not Hispanic or Latino
	Other races, not Hispanic or Latino
Housing type and transportation	Multi‐unit structures
	Mobile homes
	Crowding
	No vehicle
	Group quarters

Data on wind exposure in the contiguous US were obtained from packages hurricaneexposure (version 0.1.1) and hurricaneexposuredata (version 0.1.0) in R, with full coverage of space and time during our study period (Anderson, Schumacher, et al., 2020; Anderson, Yan, et al., 2020). Briefly, the data were generated by an exhaustive assessment of tropical cyclones recorded in the Atlantic Hurricane Database (HURDAT2) based on wind field modeling and validation against observations from weather stations (Landsea & Franklin, 2013). In line with literature (Parks et al., 2023), a county is considered exposed to a tropical cyclone in a given year if the maximum sustained wind in that county reached or exceeded the gale force (≥34 knots, or 63 km/hr) on the Beaufort scale on any given day of that year when the tropical cyclone was at the point of closest approach to that county. Given that the spatial scale of tropical cyclones substantially exceeds that of individual counties (tropical cyclone gale‐force wind fields typically extend 150–300 km from the storm center whereas US county size averages around 2,900 ${\text{km}}^{2}$) (Wang & Toumi, 2022), variations in wind speed are negligible across the average county size.

Tropical cyclone exposure data are available for full space and time coverage during 1995–2018, but SVI data are available only for 2000, 2010, and then biennially from 2014 to 2020. This highlights the necessity to incorporate various demographic covariates for our synthetic control study, as a long trajectory of pre‐exposure outcomes is not available in contrast to the settings of the traditional synthetic control method (Botosaru & Ferman, 2019; Kaul et al., 2015). Furthermore, there are two missing entries for SVI during the study period, for Clearfield County (FIPS code 42033), Pennsylvania, in 2014, and for Rio Arriba County (FIPS code 35039), New Mexico, in 2018. We excluded these two counties from our analysis because during our study period, Clearfield County experienced only one yearly exposure to tropical cyclones, and Rio Arriba County encountered zero exposure.

We obtained data on temperature and precipitation across the US for 1995–2018 from the Gridmet data set, with full time and space coverage during the pre‐exposed period of this study (Abatzoglou, 2013). GridMET data were developed by spatially interpolating meteorological observations from a variety of observatory networks using the Parameter‐elevation Regressions on Independent Slopes Model (PRISM) (Abatzoglou, 2013; Daly et al., 2008). We calculated spatially averaged monthly values of each variable across grid cells (4‐km resolution) and converted the gridded data to the county level. The summer months were defined from June to August.

We compiled all demographic and socioeconomic raw data at the county level from several public sources. Data that reflect basic demographic characteristics were obtained from CDC Wonder (United States Department of Health and Human Services (US DHHS), Centers for Disease Control and Prevention (CDC), National Center for Health Statistics (NCHS), 2021), including Percentage Black Population, Percentage Hispanic Population, Male/Female Ratio, Population Size, with full time and space coverage over the pre‐exposed period. Population density was calculated from the Population Size. Per Capita Income was obtained from the Bureau of Economic Analysis (BEA) (U.S. Bureau of Economic Analysis, 2022), and was adjusted for inflation using the year 2000 as baseline and July values derived from the Consumer Price Index (CPI) inflation calculator, according to the US Bureau of Labor Statistics (BLS) (U.S. Bureau of Labor Statistics, 2022). The Percentage Below Poverty Threshold was obtained as the Small Area Income and Poverty Estimates (SAIPE) from the US Census Bureau (U.S. Census Bureau, 2021); and the percentage of over age 25 years with a high‐school diploma was obtained from the US Department of Agriculture (USDA) as educational attainment (U.S. Department of Agriculture, 2025).

For all outcomes, exposures, and covariates, associated county identification codes were made consistent, mutually exclusive, and collectively exhaustive over time.

### 2.2 Creating Synthetic Controls

**Figure 1:**
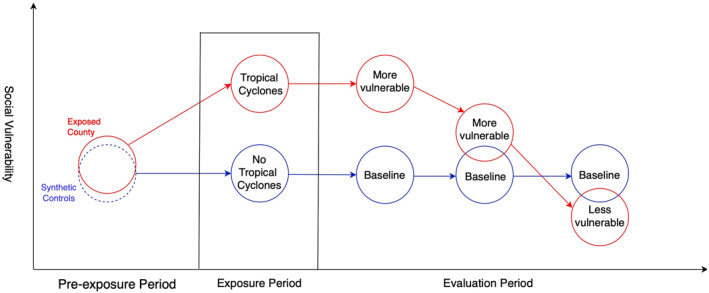
Overview of the quasi‐experimental design. Exposed and unexposed units are defined by tropical cyclone exposure within the year period. We create synthetic controls as a weighted set of unexposed counties that maintain similar trajectories on pre‐exposure social vulnerability index (SVI), tropical cyclone exposure status, and other meteorological and socioeconomic covariates. These synthetic controls are then used as counterfactuals in the evaluation period to estimate the effects of tropical cyclones on SVI.

First, we constructed the synthetic control region from a weighted set of counties in the donor pool (i.e., the full set of unexposed counties) that most closely resembled the exposed region in pre‐exposure covariates over time. Briefly, the synthetic control method is a quasi‐experimental design that aims to construct a weighted combination of unexposed units as controls to which the exposed unit is compared. This comparison is used to estimate what would have happened to the exposed unit if it had not been exposed. Here, we formed the synthetic control region that had historical trajectories of tropical cyclone exposure and SVI, as well as meteorological, demographic, and socioeconomic profiles similar to those of the exposed counties. We divided the time series of each unit into three periods (shown in Figure 1). Specifically, (a) the pre‐exposure period serves the source of calculating weights for each unexposed unit to create synthetic control units that mirrors the exposed unit as closely as possible; (b) the exposure period defines the exposure status (i.e., the exposed unit is the unit experiencing tropical cyclones in this period); (c) the evaluation period forms the outcome comparisons between the exposed unit and the synthetic control units to estimate what would have happened to the exposed unit if it had not been exposed. To ensure sufficient comparability, we balanced over 10 years of pre‐exposure covariate trajectories between the exposed regions and their synthetic controls. Table 2 shows the comparison of the pre‐exposure characteristics of the actual exposed region with those of the synthetic control regions in 2005 and 2018 (the first and last years of exposure).

**Table 2:** Covariate Means Prior to Exposures in 2005 and 2018

Covariate	2005 exposure cohort			2018 exposure cohort		
	Exposed (*n* = 217)	Unexposed (*n* = 2,998)	Synthetic control	Exposed (*n* = 303)	Unexposed (*n* = 2,912)	Synthetic control
Past TC exposure, mean	0.26	0.04	0.26	0.21	0.03	0.19
Baseline SVI, percentile	0.77	0.48	0.77	0.75	0.47	0.74
Summer temperature, °C	27.0	22.8	26.8	26.3	23.0	26.0
Summer precipitation, mm/day	4.7	2.9	4.6	4.3	3.0	4.3
High school graduate, %	71.3	77.1	71.3	75.6	81.0	75.8
Black, %	27.8	7.7	27.4	33.2	6.8	32.9
Hispanic, %	5.6	6.0	5.6	4.3	7.7	4.7
Below poverty, %	18.6	13.9	18.5	19.2	14.9	19.3
Per capita income, USD	21,724	23,636	21,693	23,643	26,154	23,680
Male‐to‐female ratio	1:1.03	1:1.02	1:1.03	1:1.02	1:1.01	1:1.02
Age <15 years, %	21.5	20.8	21.5	19.7	19.8	19.8
Age ≥65 years, %	13.8	15.0	13.9	14.6	16.0	14.7
Population density, per km^2^	149	251	149	221	259	249
Population size	115,032	87,795	117,092	75,486	97,446	79,064

*Note.* All variables except for SVI were averaged from 1995 to the year prior to the exposure year. Per‐capita income was adjusted for inflation using the year 2000 as baseline and July values.

The validity of the synthetic control approach depends on the synthetic control region being representative of the exposed region (Abadie, 2021). The key assumption underlying the causal validity of this synthetic control design is that had the exposed regions not been exposed to tropical cyclones for that particular year, their future SVI would have matched that of the synthetic control region, and as a result, any future divergence in SVI for the exposed versus synthetic control regions was attributed to tropical cyclone exposure of that year. The validity of this assumption depends on two factors: first, the availability of adequate unexposed units to build a synthetic control region capable of consistently tracking the historical trajectories of the exposed region before exposure (Abadie, 2021); and second, the absence of unobserved confounders that could cause future divergence in SVI between exposed and synthetic control regions.

**Figure 2:**
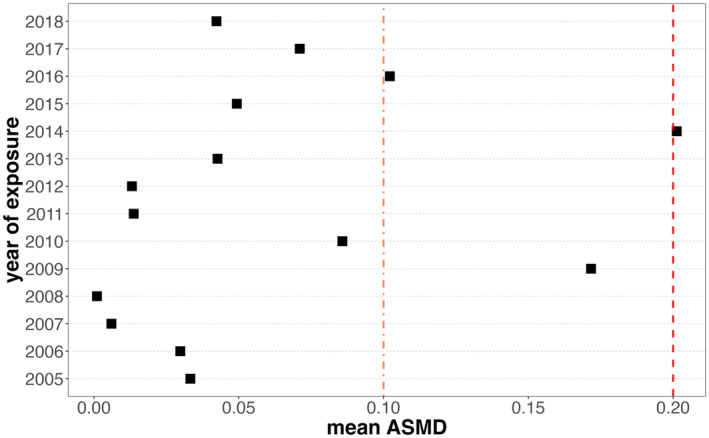
Mean absolute standardized mean difference for all covariates for exposure years.

For the first point, we assessed the quality of the synthetic control design via covariate balance to check the degree to which the distributions of pre‐exposure covariate trajectories are similar across exposure and synthetic control regions (Ho et al., 2007). Figure 2 shows the covariate balance checks throughout the pre‐exposure period for all variables and all exposure years, using mean absolute standardized mean difference (ASMD) as a metric (Parast et al., 2020). Except for three exposure years out of the 14‐year exposure period, the mean ASMD was well below 0.1, rendering our synthetic control approach effective in achieving covariate balance across a wide variety of covariates. Further sensitivity analyses also found that the main results remained almost unchanged even if we excluded these three less balanced years (detailed in Supporting Information S1). This is because only units that are similar in both observed and unobserved confounding factors are expected to exhibit comparable outcome trajectories over extended periods (Abadie, 2021). Adjusting for historical SVI trajectories allows us to account for such types of unmeasured confounders, which represents a major advantage of the synthetic control method.

### 2.3 Estimating Causal Effect

First, we formally define the causal effect of interest. Let N denote the total number of study units (i.e., counties) and T denote the total number of time points (i.e., years) in the time series. Define the year of exposure as $T_{0}$, where $1<T_{0}<T$. For unit i=1,…,N, let $D_{i,T_{0}}$ be the binary exposure status to tropical cyclones at $T_{0}$. Specifically, a county is classified as exposed if it experienced ≥1 tropical cyclone during the given $T_{0}$. Let $X_{i,t}$ be the set of pre‐exposure covariate trajectories for $\left(t<T_{0}\right)$. To ensure maximal control of covariate trajectories, $X_{it}$ includes the yearly average of pre‐exposed meteorological conditions (e.g., precipitation and summer temperature), tropical cyclone trajectory (prior exposure as defined for $T_{0}$), historical SVI, and census tract demographic variables (e.g., population density, percentage of youth and elderly, per capita income, poverty level, percentage of high school graduates, etc.). Further, let $Y_{i,t}$ be the outcome of interest, SVI, in year t during the post‐exposure period $\left(T_{0}<t<T\right)$; and $Y_{i,t}\left(d\right)$ the counterfactual outcome had the i‐th unit received exposure status d∈{0,1} in year $T_{0}$. The goal is to estimate the Average Causal Effect of Exposed (ACEE) regions for each exposure year $T_{0}$ on each outcome year t. Denote lag $\tau =t-T_{0}$, and the ACEE as

$${\text{ACEE}}_{\tau ,T_{0}}=E\left[Y_{i,T_{0}+\tau }\left(1\right)|D_{i,T_{0}}=1\right]-E\left[Y_{i,T_{0}+\tau }\left(0\right)|D_{i,T_{0}}=1\right],$$

which measures the long‐term effect of tropical cyclone exposure at time $T_{0}$ on the outcome τ years after exposure. The first term, $E\left[Y_{i,T_{0}+\tau }\left(1\right)|D_{i,T_{0}}=1\right]$, can be estimated by averaging observed outcomes from the exposed region. The second term, $E\left[Y_{i,T_{0}+\tau }\left(0\right)|D_{i,T_{0}}=1\right]$, represents a counterfactual quantity, and its estimation requires the construction of synthetic controls.

We estimated the causal effect of annual tropical cyclones on SVI in subsequent years by iteratively constructing synthetic controls for the exposed region corresponding to each exposure year during 2005–2018. For the exposure year 2009, as an example, we constructed synthetic control regions using pre‐exposure covariate trajectories during 1995–2008 and computed the ACEE for each outcome year‐2010, 2014, 2016, 2018, and 2020‐when the SVI data were available.

### 2.4 Pooling Estimates

To investigate long‐term effects, we examined post‐exposure periods ranging from one to 15 years after each exposure year, allowing us to study the lagged effect of tropical cyclone exposures for up to 14 years of lag. The synthetic control approach described above can be used to produce ${\text{ACEE}}_{\tau ,T_{0}}$ estimates for each pair of year lag and exposure year $\left(\tau ,T_{0}\right)$, representing the effects of tropical cyclones on exposure year $T_{0}$ on SVI of the affected regions τ years later. For years in which many counties were exposed to tropical cyclones, these individual ${\text{ACEE}}_{\tau ,T_{0}}$ estimates may be useful on their own; however, in other years, these estimates were too noisy to be interpreted on their own. As such, we pooled the estimated ${\text{ACEE}}_{\tau ,T_{0}}$s’ at the same lag from different exposure years. We built Wald‐type CIs based on the standard errors (SE) estimated using a Jackknife variance estimator clustered at each outcome year level (Efron, 1982). That is, we pooled the individual estimates of the ${\text{ACEE}}_{\tau ,T_{0}}$‐defined in Section 2.3 and estimated via the synthetic control method‐across different values of the exposure year $T_{0}$ and lag τ through natural splines modeling:

$${\text{ACEE}}_{\tau ,T_{0}}=\beta_{0}+\beta_{1}\cdot \text{ns}\left(\tau ,df=k\right)+\epsilon ,$$

which was considered due to its flexibility in capturing non‐linear trajectories in vulnerability (e.g., an initial spike in vulnerability followed by long‐term decline). The natural splines model captured two assumptions: first, that the effect of tropical cyclone exposure on SVI did not depend on the start year $T_{0}$ and that the effect tended to zero with time. Clearly, as the lag tends to infinity, any effects of prior tropical cyclone exposures on SVI must vanish, that is, $\lim_{\tau \to \infty }{\text{ACEE}}_{\tau ,T_{0}}=0$, and so the above equation must fail for large values of τ. However, this model fitted our data well in the estimation range, τ∈[1,14]. Further, to determine the complexity of the model based on the data, the degrees of freedom k were selected by optimizing the average of the Akaike Information Criterion (AIC) and the Bayesian Information Criterion (BIC). While the AIC favors more complex models with better predictive accuracy, the BIC penalizes extra parameters based on sample size and favors simpler models. By taking an average of the two, we strive to select the optimal models with predictive accuracy while preventing overfitting. Point‐wise CIs for ${\text{ACEE}}_{\tau ,T_{0}}$ were obtained through a Wald‐type construction with jackknife standard errors, which is a robust variance estimator especially for small samples that estimates uncertainty by systematically removing one cluster of observations at a time and recalculating the effect (Efron, 1982). To account for the fact that tropical cyclone exposure can be highly idiosyncratic across years, and a given county might‐for a given outcome year‐serve both as a treated unit for one lag and contribute to the synthetic control for a different lag, the jackknife was clustered at the outcome year level (i.e., all observations with the same value of $t=T_{0}+\tau$ were considered to be in the same cluster for the purpose of jackknifing).

### 2.5 Placebo Test

The synthetic control method allows inferential procedures based on placebo tests (Abadie et al., 2010). Similar to the framework for permutation tests, where the distribution of a test statistic is computed under random permutations of the sample units' assignments to the treated and control groups, the in‐space placebo test uses placebo, that is, “fake treatment units” for statistical inference. We applied the following hypothesis testing:

$$H_{0}:\Delta =0\;\text{v.s.}\;H_{a}:\Delta \neq 0,$$

where Δ denotes the overall rate of change in SVI throughout the entire study period. The overall causal effect is considered significant if the estimated effect was unusually extreme relative to the distribution of placebo effects. Specifically, for each year of exposure (2005–2018), denote the number of exposed units (i.e., counties) as $n_{1}$ and define the donor pool as the units that were not exposed to tropical cyclones at all during 2005–2018. For each iteration i (50 for each exposure year), we selected at random $n_{1}$ units from the placebo pool, assuming that they were assigned a fake tropical cyclone exposure. The donor pool for the fake exposed units (placebo units) includes all other units in the data set except for the placebo units; then, for the placebo units, we found the synthetic control units and calculated their respective weights. Finally, we calculated the population‐weighted difference in SVI between the fake exposed and synthetic control regions for each outcome year. We took averages of iterations for each exposure year, refitted the outcome model, and built the CIs for the pooled estimates. Finally, we performed hypothesis tests on the distribution of Δ obtained from each placebo run. Under $H_{a}$, we used a two‐sided *p*‐value defined as the frequency that the absolute values of the placebo effects were greater than or equal to the absolute value of the estimated change from real data:

$$p‐\text{value}=\frac{1}{N+1}\sum_{i=1}^{N+1}I\left(\left|{\hat{\Delta }}_{i}|\geq |\Delta_{1}\right|\right),$$

where ${\hat{\Delta }}_{i}$ denotes the estimated placebo effect for placebo run i, and $\Delta_{1}$ the estimated causal effect from real data. This procedure was repeated for the overall SVI and for each subdomain of the SVI. The natural splines model for each outcome informed by the optimal BIC and AIC had a df=1 except for the SVI of Minority Status/Language domain, which gave an optimal df=2. A linear model was interpolated to calculate Δ and obtain p‐values.

## 3 Results

**Figure 3:**
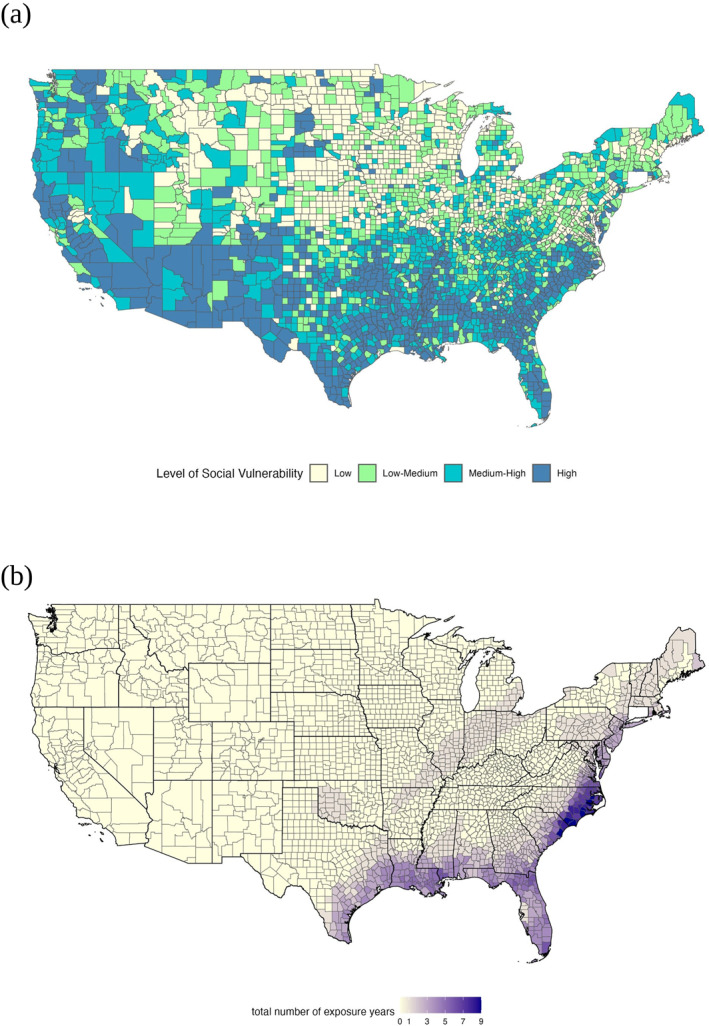
Overview of exposure and outcome variable. (a) Social vulnerability index by US county in 2020; (b) Number of years with tropical cyclone exposure by county for 2005–2018.

Many of the nation's most socially vulnerable communities were concentrated in southern coastal regions, particularly the Gulf Coast and the Southeastern US (Figure 3a). These regions of high SVI overlapped substantially with counties frequently affected by tropical cyclones (Figure 3b). Between 2005 and 2018, 47 named Atlantic basin tropical cyclones resulted in 2,118 annual tropical cyclone exposures in 948 counties. Six of the seven counties with the highest annual counts of tropical cyclone exposure during this period were in North Carolina, each experiencing tropical cyclones in nine of the 14 years studied.

**Figure 4:**
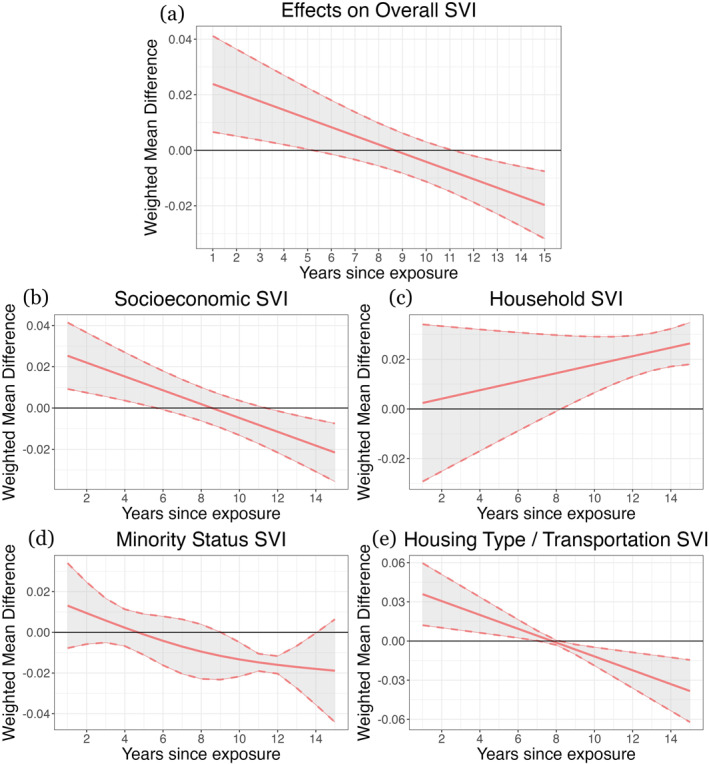
Effects of tropical cyclone exposures on community social vulnerability up to 15‐year lags, pooled across exposure years 2005–2018. Two‐sided 95% CIs are presented.

Counties affected by tropical cyclones exhibited a 2.4% [95% Confidence Interval (CI): 0.7%–4.1%] increase in SVI 1 year after exposure, with this increase persisting for at least 5 years (Figure 4a). However, over time, SVI trended downward throughout the post‐exposure period and eventually fell below the baseline levels of their unexposed counterparts after 12 years, indicating a decrease of −1.0% [95% CI: −1.9%–0.0%] in SVI. Finally, 15 years after tropical cyclone exposure, the SVI of the exposed regions was −2.0% [95% CI: −3.2% to −0.8%] lower than the baseline.

Among the four subdomains of SVI (Figures 4b–4e), the Socioeconomic and Housing Type and Transport subdomains contributed the most to this consistently downward trend in the overall SVI. For the Socioeconomic domain, the trajectory closely aligned with that of the overall SVI. One year after tropical cyclone exposure, the Socioeconomic SVI increased by 2.5% [95% CI: 0.9%–4.1%] compared to baseline, persisting for 5 years. This initial spike attenuated in subsequent years and eventually fell below the baseline in year 12. By year 15, the exposed counties showed an average decrease in SVI of 2.2% [95% CI: −3.6% to −0.7%] compared to baseline. A similar trend can be observed for the Housing Type and Transportation domain, with SVI increasing to 3.6% [95% CI: 1.2%–6.0%] 1 year after exposure to tropical cyclones. The increase persisted, but diminished at the end of seven years, crossing below baseline by year nine and declining to −3.8% [95% CI: −6.2% to −1.5%] by year 15. For the domain of Minority Status and Language Proficiency, the overall trend was downward, with an initial SVI increase of 1.3% [95% CI: −0.8%–3.4%] one year post‐tropical cyclones and a decrease to −1.9% [95% CI: −4.4%–0.6%] by year 15. However, CIs encompassed zero except for years 9–14 after exposure, when exposed counties showed significantly lower SVI compared to baseline levels of their unexposed counterparts.

The SVI of the Household Characteristics subdomain increased from 0.2% [95% CI: −2.9%–3.4%] 1 year after tropical cyclones to 2.6% [95% CI: 1.7%–3.5%] by year 15, indicating a sustained increase in the Household SVI over time, which differed from the trends in other subdomains. As such, we tracked the estimated trajectories for the individual factors that made up this domain. In particular, we found a gradual decrease in the percentage of older adults over 65 years of age and an increase in the percentage of young people under 17 years of age, as well as an increase in the percentage of the disabled population among regions affected by tropical cyclones, indicating a demographic shift in age and disability structure (details in Supporting Information S1).

**Figure 5:**
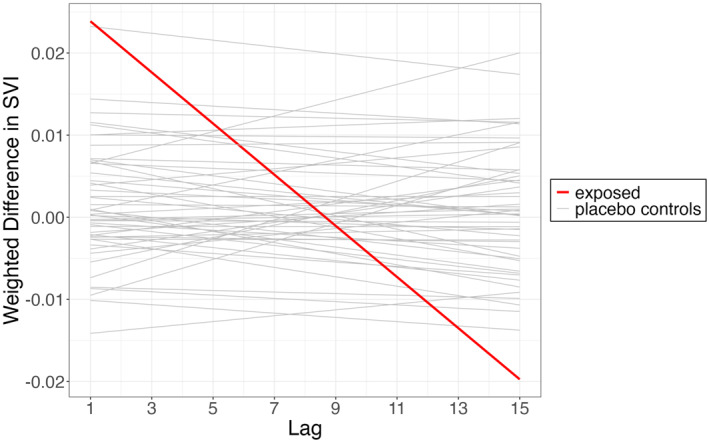
Long‐term effects of social vulnerability index in exposed counties and placebo effects in all 50 placebo runs.

Figure 5 shows the results of the placebo tests, in which we compared the observed effects with the placebo effects obtained by treating a randomly selected set of unexposed units as if they were exposed. The SVI trend for counties truly exposed to tropical cyclones was significantly larger relative to the distributions of the estimated trends for the units in the placebo pool. The two‐sided hypothesis test on the slope of the overall change in SVI yielded a *p*‐value of <0.02, which indicated the statistical significance of the observed effects. Similar results were obtained for some SVI subdomains, namely the Socioeconomic, Minority Status and Language, and Housing Type and Transportation domains, each with a *p*‐value of <0.02. The only exception was the domain of household characteristics, which had a *p*‐value of 0.12, rendering it insignificant at the α=0.05 level. However, as shown in Text 3.4 and Figure S20 in Supporting Information S1, some variables within this domain, such as the percentage of older adults and young people, did exhibit statistically significant shifts (p<0.05), suggesting that the aggregate index may mask important underlying demographic transitions.

## 4 Discussion

In this study, we evaluated the evolution of social vulnerability after exposure to tropical cyclones by counterfactually comparing counties that encountered tropical cyclones with unexposed control counties whose past attributes closely match those of exposed counties. The main findings of the results are twofold: (a) it confirmed the adverse effects of tropical cyclones over a relatively short period of time, intensifying social vulnerability for many, though not all, perspectives of society, consistent with the literature (Parks et al., 2023; Yabe et al., 2020); (b) it revealed a turning point in the effects of tropical cyclone exposure over a longer period of time. Our findings align with a previously documented hypothesis (Crespo Cuaresma et al., 2008; Hallegatte & Dumas, 2009), which suggests that affected regions may initially suffer, as lives may be lost and productive capital destroyed. However, gradual replacement of lost assets with modern units has a positive net effect on long‐term growth. The existence of gentrification may have contributed to these affected regions becoming less vulnerable over time, even compared to their unexposed counterparts. However, this positive net effect can come at the cost of justice, as the long‐term socioeconomic impacts of tropical cyclones, if left unaddressed, could function as a form of disaster‐driven gentrification (Fussell, 2015).

A long‐term positive shift in regional social vulnerability does not necessarily indicate improvements for the people most affected by tropical cyclones. Instead, this apparent improvement may be driven by population shifts following tropical cyclones, rather than by improved conditions for the people who suffered the most. Climate gentrification is a well‐described phenomenon in which the effects of climate hazards lead to changes in the social and physical fabric of affected regions (Anguelovski et al., 2019; Freeman, 2005). The literature has found that low‐income communities, people of color, and migrant communities are more likely to experience residential and social displacement, but at the same time, rebuilding efforts often attract an influx of wealthier residents and increased investment in affected areas after major hurricane disasters (Aune et al., 2020; Greenberg, 2014; Van Holm & Wyczalkowski, 2019). Recently, a spatial analysis of Orleans Parish after Hurricane Katrina found significant racial turnover as investment flowed to safer, higher‐elevation neighborhoods, displacing the original low‐income, predominantly Black communities (Aune et al., 2020). Our findings echo the direction of these shifts, showing a statistically significant, albeit small, decreasing trend in Minority Status SVI (Figure 4d) over years 9–14 post‐exposure, suggesting that the ethnic composition of affected communities gradually shifts over time, potentially serving as a broad quantitative indicator of gentrification.

Our study is a large‐scale quantitative investigation that provides evidence of a statistically significant relationship between tropical cyclone exposures and SVI, with three major strengths. First, we focused on the long‐term impacts on composite social vulnerability rather than on a single measure of economic or health outcomes, thus elucidating the evolution of social vulnerability after tropical cyclones as a potential root cause of economic or health consequences. Second, our results provide evidence for a causal link between tropical cyclone exposures and community social vulnerability by employing the synthetic control methods, which is an extension of the difference‐in‐differences approach that controls time‐varying measured confounders (Wu et al., 2023) and time‐invariant unmeasured confounders (Arkhangelsky et al., 2021; Bouttell et al., 2018). Third, our work provides a methodological framework that integrates big data with advanced statistical methods to improve our understanding of the impacts of extreme weather events.

Our study also has several limitations. A primary consideration is that the CDC/ATSDR SVI is an approximate and imperfect measure of the theoretical construct of social vulnerability (Bakkensen et al., 2017; Spielman et al., 2020; Tate, 2012). While the SVI has faced criticism regarding its validation (Rufat et al., 2019, 2020), it remains a widely used tool (Centers for Disease Control and Prevention Agency for Toxic Substances Disease Registry, 2025; Painter et al., 2024), and recent validation efforts have confirmed its favorable performance in disaster contexts (Freelander et al., 2024; B. Flanagan et al., 2019). We also note that while the SVI methodology underwent minor nationwide updates during the study period (e.g., 2010, 2020), these secular changes affect both exposed and control units equally and are thus accounted for by the comparative nature of the synthetic control design (Abadie, 2021). Another potential limitation stems from the SVI's reliance on 5‐year ACS data, which may create a potential temporal overlap between exposure and outcome variables. To address this, we performed a sensitivity analysis by adjusting the exposure windows to eliminate this overlap and confirmed that our results remained robust. Future work should focus on new methods in the synthetic control method that incorporate multiple outcomes to improve power and precision (Sun et al., 2025). Furthermore, another potential limitation of our analysis is the risk of temporal unmeasured confounding bias inherent in observational data, which makes it difficult to disentangle the specific impact of tropical cyclones from other concurrent temporal changes affecting SVI. However, we conducted extensive placebo tests and sensitivity analyses to assess the robustness of our estimates against spurious associations. Post‐cyclone migration could also introduce interference effects between geographic units (Tchetgen & VanderWeele, 2012); however, our county‐level analysis is intended to capture the net effect of population shifts on the aggregate vulnerability of exposed regions, thus reducing concerns about individual‐level interference. Finally, this study uniformly assessed tropical cyclones without differentiating by intensity, duration, or frequency of exposure. To address this, we presented a stratified analysis in Supporting Information S1 for hurricane‐force exposure, showing that higher‐intensity storm exposure is likely the principal driver of heightened vulnerability post‐disaster. Future methodological work should look to expand synthetic control methods to incorporate continuous exposures, which remains methodologically challenging.

In summary, our analyses offer new insight into how social vulnerability changes after tropical cyclones. The results of our research further contextualize how disaster responses can be more effective and how communities can be more resilient, which is a matter of environmental and social justice. Although tropical cyclones are not selective of the communities they impact, the characteristics of communities play an important role in their protection and resilience. Residents in socially vulnerable communities are expected to be differentially impacted during all periods around the natural disasters: pre‐disaster communication and preparation, emergency response, recovery, and reconstruction. Residents in these affected communities often bear the greatest burden, yet are not among those who benefit from resource allocation in disaster response efforts. Post‐disaster displacement and resettlement of original residents could decrease access to healthy foods, transportation, healthcare, and education and increase injuries, violence, and crime while disrupting community cohesion (Aune et al., 2020; Smith et al., 2020). When disasters cannot be ameliorated with precisely allocated resources, they can lead to exaggerated social disparities and amplify the adverse impacts of tropical cyclones. This research emphasizes the importance of equitable long‐term planning from the social and environmental justice perspectives, highlighting its role in significantly reducing the long‐term impacts of tropical cyclones on human suffering (Cardwell & Konrad, 2022; Griego et al., 2020; Willison et al., 2019). Social vulnerability factors, such as limited economic opportunities, inadequate transportation systems, and inequitable housing conditions, can undermine the resilience of a community to disaster‐related suffering. Furthermore, exposure to tropical cyclones can alter the social vulnerability of affected communities in both anticipated and unexpected ways.

Moving forward, it is critical to address these complexities through stratified analysis by storm intensity and frequency, which would help identify the specific thresholds that trigger changes in community vulnerability and to understand the compounding effects of multiple exposures over time. To overcome inherent challenges with vulnerability measures and to make more robust conclusions, future work should integrate multiple vulnerability measures and make use of advanced techniques in causal inference in panel data. Finally, more granular analyses on specific categories of social vulnerability–perhaps through targeted case studies–will reveal localized dynamics that are masked at the county level. Through present and future work, we could better understand the social and health disparities faced by communities affected by tropical cyclones and inform targeted interventions that promote equitable recovery in the aftermath of tropical cyclone events.

## Conflict of Interest

XW has been employed at Meta since July 2025, though the work presented in this manuscript was conducted prior to joining the company. The remaining authors declare no competing interests.

## Supporting information

Supporting Information S1

## Data Availability

All data sets utilized in this study are publicly available. The repositories for the processed data are listed in Table S1 in Supporting Information S1. The CDC/ATSDR SVI data were obtained from CDC/ATSDR (Centers for Disease Control and Prevention Agency for Toxic Substances Disease Registry, 2025); data on wind exposure in the contiguous US were obtained from the hurricaneexposuredata R package (Anderson, Schumacher, et al., 2020), which derives county‐level wind estimates from the HURDAT2 database (Landsea & Franklin, 2013); data on temperature and precipitation were obtained from PRISM Group (PRISM Group, 2024); demographic data including percentage black, percentage hispanic population, male/female ratio, and population size were obtained from CDC Wonder (United States Department of Health and Human Services (US DHHS), Centers for Disease Control and Prevention (CDC), National Center for Health Statistics (NCHS), 2021); per capita income was obtained from BEA (U.S. Bureau of Economic Analysis, 2022) and adjusted for inflation using the year 2000 as a baseline using July values derived from the Consumer Price Index (U.S. Bureau of Labor Statistics, 2022); percentage below poverty threshold were obtained from the U.S. Census Bureau (U.S. Census Bureau, 2021); and percentage of over age 25 years with high‐school diploma was obtained from the U.S. Department of Agriculture (U.S. Department of Agriculture, 2025). The code used for the analyses and the data shown in the figures are publicly available at https://github.com/LincoleJ/tropical_cyclone_svi.git (Jiang et al., 2026).
