# Supplementary material for: Evolution of Long‐Term Social Vulnerability After Tropical Cyclones in the United States
Source: Geohealth. 2026 Jul 24;10(7):e2025GH001727. doi: 10.1029/2025GH001727 (PMC13400701; doi:10.1029/2025GH001727)
Supplement: Supplementary file 1 — Supporting Information S1 [file GH2-10-e2025GH001727-s001.pdf]

Supplementary Information: Evolution of Long-Term Social Vulnerability After Tropical Cyclones in the United States

Lingke Jiang[1], Robbie M. Parks[2*], Yoshira Ornelas Van Horne[3], Xiao Wu[1*]

[1*]Department of Biostatistics, Columbia University Mailman School of Public Health, 722 W 168th St, New York, 10032, NY, USA.
[2]Department of Environmental Health Sciences, Columbia University Mailman School of Public Health, 722 W 168th St, New York, 10032, NY, USA.
[3]Department of Environmental Health Sciences, UCLA Fielding School of Public Health, 650 Charles E Young Dr S, Los Angeles, 90095, CA, USA.

*Corresponding author(s). E-mail(s): rmp2198@columbia.edu; xw2892@cumc.columbia.edu;
Contributing authors: lj2575@cumc.columbia.edu; yoshira@g.ucla.edu;

Contents

1 Additional Analyses **3**

1.1 Hurricane Sandy: a Case Study . . . 3

1.2 Evolution of Long-term Social Vulnerability after Hurricanes . . . 3

1.3 Descriptive Long-term Trends of Social Vulnerability . . . 4

2 Additional Data Descriptions **4**

3 Additional Analytic Details **5**

3.1 Covariate Balancing for Synthetic Control . . . 5

3.2 Covariate Balance Assessment . . . 6

3.3 Pooled Estimates . . . 8

3.4 Placebo Tests Results on Domains of SVI . . . 8

3.5 Additional Analysis on Individual Variables of Minority Status / Language Proficiency SVI . . . 9

4 Sensitivity Analyses **9**

4.1 Outcome Analysis Excluding Poorly Balanced Exposure Years . . . 10

4.2 Outcome Analysis with Strictly Non-overlap Between Exposure and Outcome Data Sources . . . 10

5 Software **11**

1 Additional Analyses

Our study presents an aggregated effect of tropical cyclone-force exposure on community social vulnerability over the long term, and observe a gradually decreasing effect. Here, we illustrate the underlying drivers behind this phenomenon by first, studying the aggregated effect of hurricane-force winds on long-term social vulnerability; illustrate how this plays out for individual storms via a case study of Hurricane Sandy to illustrate post-cyclone gentrification; and present a descriptive analysis of the evolution of social vulnerability.

1.1 Hurricane Sandy: a Case Study

To illustrate how the aggregate trend is reflected on individual counties, we conducted a case study on counties exposed to Hurricane Sandy in 2012. We tracked the evolution of long-term vulnerability of these counties, compared to their weighted synthetic control units, which were constructed from the donor pool of unexposed units across contiguous US in 2012, consistent with the main analysis. Fig. S1 shows the trajectory of overall SVI for exposed counties (solid red line) as opposed to the synthetic controls (dashed blue line). Prior to the exposure (2000-2012), the trajectory of the synthetic control closely tracked the exposed counties and showed pre-exposure alignment. Immediately following the event in 2012, we observe elevated social vulnerability in the exposed counties relative to the synthetic controls. However, this trend started to reverse in 2018, and by 2020, the exposed counties exhibited a notably lower vulnerability compared to the counterfactual. These results align with the findings of our main analysis and corroborate existing literature that detail the gentrification post-Sandy [1].

1.2 Evolution of Long-term Social Vulnerability after Hurricanes

To investigate the causal effect on social vulnerability driven by hurricane-force ($\geq$ 64 knots) exposure, we performed the same synthetic control and pooled outcome analysis for hurricane exposure. Here, exposure is defined for a given county if for a given year, the maximum sustained wind in that county reached or exceeded hurricane force ($\geq$ 64 knots, or 32.92 m/s on the Beaufort scale on any given day of that year when the tropical cyclone was at the point of closest approach to that county). In contrast, the donor pool is defined as the counties not exposed to tropical cyclone-force at all during that particular year. Due to the rarity of hurricane exposures, we focus this analysis on four years with significant hurricane activity (exposed counties $\geq$ 10): 2005, 2008, 2017, and 2018. As shown in Fig. S2, the analysis shows a consistent recovery trend where vulnerability initially spikes then gradually decline over time. However, the magnitude of the heightened vulnerability is much more pronounced for hurricane exposure compared to the aggregate analysis. Specifically,

overall SVI increased by approximately 17.3% one year after hurricane exposure, nearly 7 times compared to the 2.4% increase observed for tropical cyclone exposure. Overall, this stratified analysis suggests that the aggregated increase in social vulnerability observed in the main analysis is disproportionately driven by hurricane events, compared to tropical storms with lower intensity.

1.3 Descriptive Long-term Trends of Social Vulnerability

To complement the causal design presented in Fig. 4 of the main text, we examine the unadjusted descriptive trends of social vulnerability index. As shown in Fig. S3, exposed counties consistently exhibit higher baseline SVI trends compared to the unweighted donor pool. Despite the persistent gap between the SVI values of the exposed units and that of the unweighted donor pool, the synthetic control units successfully minimizes this pre-exposure imbalance. Notably, despite a general increase in SVI observed in 2020 across the exposed cohorts, the synthetic control units closely mirror this trend, which indicates that the synthetic control design effectively captures structural temporal changes or external shocks affecting the measurement scale, consistent with literature [2]. Furthermore, while individual exposure years (e.g., 2006 and 2012) suggest a trend of decreasing vulnerability post-exposure, the trajectories exhibit significant noise. This variability underscores the necessity of the pooled analysis via natural splines modeling used in the main text to robustly estimate the average causal effect.

2 Additional Data Descriptions

Table S1 summarizes the publicly available data sources used for this study. All data were collected with annual temporal resolution and county-level spatial resolution. The Federal Information Processing Standard (FIPS) codes were made consistent over time to account for changes in geographic boundaries, the creation or deletion of counties or jurisdictions, and name changes for counties or cities. In Table 2 of the main text, we display the comparison between the pre-exposure characteristics of the actual exposed region with that of the unexposed regions in 2005 and 2018, corresponding to the covariate and pre-exposure outcome characteristics in the first and last exposure year over our study period. In Tables S2-S13, we show the comparisons of all other exposure years during 2006-2017. From these comparisons, exposed counties generally had much higher past tropical cyclone exposures and roughly 5% to 36% higher social vulnerability prior to tropical cyclone exposure compared to the crude average of all unexposed counties (i.e., the donor pool), suggesting very different covariate profiles of exposed versus unexposed regions before applying the synthetic control method.

3 Additional Analytic Details

3.1 Covariate Balancing for Synthetic Control

A key aspect of the synthetic control method is ensuring that the synthetic control units are similar to the exposed units in pre-exposure covariate trajectories. We achieved this by casting our synthetic control problem with extended balance conditions as a Covariate Balancing Propensity Score (CBPS) problem using unconstrained convex optimization [3]. For the initial implementation, we treated all exposed units in a given exposure year T_0 as an aggregate average unit of all exposed units. For example, assuming that the analysis is based in exposure year 2009, denote the number of exposed units by n_1 and the number of unexposed units as $n_0 (= N - n_1)$. With the d-dimensional covariates $\mathbf{X}_{i,t} \in \mathbb{R}^d$, we created a vector $\mathbf{X}_i$ that horizontally stacked the time-varying covariates $\mathbf{X}_{i,t}$, which characterize the complete trajectory of a unit during the pre-exposure period (1995-2008):

$$\mathbf{X}_i = [\mathbf{X}_{i,1}, ..., \mathbf{X}_{i,t}] \quad (t < T_0).$$

Our objective was to obtain a set of weights $w_i \in \mathbb{R}$ such that the average covariate trajectory of the exposed units roughly equals to that of a weighted set of control units:

$$\frac{1}{\sum_{i=1}^{n} \mathbf{1}\{D_{i,T_0} = 1\}} \sum_{i:D_{i,T_0}=0} w_i \mathbf{X}_i \approx \frac{1}{\sum_{i=1}^{n} \mathbf{1}\{D_{i,T_0} = 1\}} \sum_{i:D_{i,T_0}=1} \mathbf{X}_i,$$

where $\approx$ denotes an approximate equality between the multi-dimensional vectors. These weights are given by formulating the connection between the propensity score weighting and the balancing,

$$w_i = \frac{e(x;\theta)}{1 - e(x;\theta)},$$

where $e(x;\theta)$ denotes the propensity score of the exposure:

$$\frac{1}{\sum_{i=1}^{n} \mathbf{1}\{D_{i,T_0} = 1\}} \sum_{i:D_{i,T_0}=0} \frac{e(x;\theta)}{1 - e(x;\theta)} \mathbf{X}_i = \frac{1}{\sum_{i=1}^{n} \mathbf{1}\{D_{i,T_0} = 1\}} \sum_{i:D_{i,T_0}=1} \mathbf{X}_i.$$

We further posited a logistic regression model for the propensity score, $e(x;\theta) = 1/(1 + e^{-x\theta})$. We substituted this into the above expression and derived

$$\frac{1}{\sum_{i=1}^{n} \mathbf{1}\{D_{i,T_0} = 1\}} \sum_{i:D_{i,T_0}=0} e^{\mathbf{X}_i \theta} \mathbf{X}_i = \frac{1}{\sum_{i=1}^{n} \mathbf{1}\{D_{i,T_0} = 1\}} \sum_{i:D_{i,T_0}=1} \mathbf{X}_i,$$

which serves as the Karush–Kuhn–Tucker (KKT)-condition for the multi-dimensional convex problem:

$$\hat{\theta} = \arg\min_{\theta}\{\frac{1}{n}\sum_{i=1}^{n} l_\theta(\mathbf{X}_i, D_{i,T_0})\}, \quad l_\theta(\mathbf{X}_i, D_{i,T_0}) = (1 - D_{i,T_0})e^{\mathbf{X}_i \cdot \theta} - D_{i,T_0}\mathbf{X}_i \cdot \theta.$$

By gradient descent on the above equation, we obtained a unique solution. In practice, due to class imbalance and near separability (n_1 is very small compared to N), we solved for $l_\theta(\mathbf{X}_i, D_{i,T_0})) + \lambda|\theta|_2^2$ for some appropriately chosen λ based on minimal ASMD, taking into consideration that intervention bias is positively correlated with ASMD [4].

An important advantage of the covariate balancing weighting approach used in the analysis is that it enables assessing the quality of the approach by evaluating the covariate balance of the weighted data [4, 5]. In this study, covariate balance was assessed by estimating the average differences between the covariates of the exposed units and that of the synthetic control units divided by the standard deviations of the covariates (i.e., SMD).

3.2 Covariate Balance Assessment

The validity of the synthetic control method depends on the weighted synthetic controls being representative of the exposed region. In scenarios in which pre-exposure outcome variables are not available for an extended period of time prior to exposure, a close match of covariates that have a large predictive power on the outcome of interest was of crucial importance for constructing effective synthetic control units [6, 7]. In the traditional literature on the synthetic control method, the quality of the synthetic control estimator is typically assessed by a numerical comparison between the covariate means of the exposed regions, that of the donor pool, and the weighted covariate means of the synthetic control regions [2, 8]. This is shown in Tables S2-S13, exposed versus unexposed regions had very different covariate profiles before applying the synthetic control method. By design, the synthetic control region was constructed to resemble the exposed counties for all pre-exposure covariates, and reassuringly, the synthetic control regions achieved very comparable covariate profiles to the exposed regions. However, although common, this practice does not establish an objective assessment of the covariate balance between the exposed and synthetic control regions [4, 9]. We further assessed the similarity between exposed and synthetic control units derived from the CBPS weighting approach by evaluating the balance of covariates using the absolute standardized mean difference (ASMD), which provides an objective way of determining the goodness of fit [4, 5]. ASMD is defined as the absolute difference between the value of a specific covariate for the exposed unit and the weighted mean of that covariate in the synthetic control group, divided

by the standard deviation of the covariate within the synthetic control group. That is,

$$ASMD_{X,t} = \frac{|X_{exposed,t} - X_{SC,t}|}{sd(X_{SC,t})},$$

where $X_{exposed,t}$ denotes the covariate of the exposed unit in the pre-exposure years, $X_{SC,t}$ the weighted covariate in the synthetic control in the pre-exposure years, and $sd(X_{SC,t})$ the weighted standard deviation of the covariate in the synthetic control group in the pre-exposure year. To examine the summary of ASMD values across years, we used the mean ASMD for each covariate, which is defined as

$$\overline{ASMD}_X = T_0^{-1} \sum_{t}^{T_0} \frac{|X_{exposed,t} - X_{control,t} \cdot w|}{sd(X_{SC,t})},$$

where T_0 denotes the year prior to exposure, and w the vector of synthetic control weights.

We first applied thresholds of < 0.2 and < 0.1 to the ASMD to indicate acceptable covariate balance for each exposure year. The main analysis accounted for up to $d = 303$ pre-exposure covariates, including historical SVI and tropical cyclone exposure, meteorological profiles, and demographic characteristics. In the main text, we showed that most exposure years have mean ASMD below the 0.1 threshold across covariates, except for one exposure year; and all exposure years were below the 0.2 threshold. Specifically, we found that without using synthetic control weights, the exposed and unexposed regions were highly imbalanced with approximately 10%-65% of covariates above the threshold. After applying the CBPS algorithm, most covariates fell below the threshold.

In addition, we visually assessed the covariate balance for each individual covariates across the exposure years from 2005 to 2018. The left panels of Fig. S4-S17 show the standardized mean differences (SMD) for the average of each covariate in each exposure year. We found that most covariates in the pre-exposure period were largely imbalanced in the original unweighted data, indicating a substantial risk of confounding bias. However, after implementing the synthetic control method, covariate balance improved considerably. While these results suggest that the synthetic control method effectively improved covariate balance, concerns about potential residual confounding bias from imbalanced years remain [4]. To address this, we performed a sensitivity analysis that excluded the exposure years with imbalanced ASMD (see details in Sec. 4.1).

The right panels of Fig. S4-S17 show the geographic distributions of the exposed regions and their corresponding synthetic control regions for each year of exposure. In principle, synthetic control units must be drawn from a donor pool of potential control units similar to the exposed regions [2]. This principle was upheld, as the majority of synthetic control units that were assigned substantial weights were in the nearby regions of the exposed units, demonstrating the geographic similarity between the exposed region and the constructed synthetic control regions.

3.3 Pooled Estimates

The synthetic control method generated ACEE_{τ,T_0} estimates for each pair of lag year and exposure year (τ, T_0), which quantify the effects of tropical cyclone exposure in year T_0 on SVI τ years later. In years of many tropical cyclone exposures, these individual estimates offer valuable insights on their own. However, in most other years with only a few tropical cyclone exposures, these estimates become less reliable and harder to interpret due to noise and variability inherent to the limited number of data.

Fig. S18 displays individual ACEE_{τ,T_0} estimates for each pair of exposure year T_0 and lag τ, with each individual estimate scaled to reflect the relative population size of the exposed regions in the year of exposure. For example, the 2005 hurricane season was particularly devastating, impacting 263 counties and a population of approximately 131,000. Among the tropical cyclones that contributed to these exposure, Hurricane Katrina was the most infamous, causing nearly 1700 direct deaths and over $100 billion in damages across the US [10]. To assess the impact of this exposure, we compared the post-exposure SVI outcomes of the regions exposed to tropical cyclones in 2005 with their syntheic control regions. In 2016, 11 years after the 2005 tropical cyclone events, the exposed regions had an average SVI of 74.7%, while the synthetic control regions, which were not exposed to tropical cyclones in 2005, had an average SVI of 78.0%. This difference yields an $\text{ACEE}_{11,2005}$ estimate of -3.3%. In contrast, the 2009 hurricane season in the Atlantic basin was relatively mild, contributing to only six tropical cyclone exposures to US counties. As such, the same procedure comparing these 11-year estimates resulted in much noisier ACEE estimates. In 2020, 11 years after the 2009 exposures, the average SVI of the exposed regions was 79.2%, whereas that of the synthetic control regions was 60.2%, resulting in a noisy $\text{ACEE}_{11,2009}$ of 18.9%. For reference, the average ACEE at $\tau = 11$ given by the natural splines model was -0.7% [95% CI: -1.5% to 0.0%].

To improve power and interpretability, we pooled ACEE_{τ,T_0} estimates across different values for the exposure year using natural splines modeling for exposure year T_0 and lag τ. The resulting pooled estimate is shown as the thick black curve in Fig. S18.

3.4 Placebo Tests Results on Domains of SVI

The synthetic control method allows for inferential procedures based on placebo tests. Fig. S19 presents the results of the placebo tests for each SVI subgroup. Consistent with p-values reported in the main text–indicating that, except for the Household domain, the overall changes in outcome were statistically significant compared to the placebo runs–we observed a clear distinction between the results from the actual data (represented by the blue line) compared to the placebo runs (represented by the gray lines).

Placebo tests for individual Household Characteristics variables in Fig. S20 revealed heterogeneous effects masked by aggregation: percentage of older adults (age 65 years and above) rankings showed significant decline (p = 0.04), while percentage of young people (under age 17 years) and percentage disabled showed significant increases (p = 0.02). Single-parent household rankings showed no significant upward or downward trend (p = 0.84).

3.5 Additional Analysis on Individual Variables of Minority Status / Language Proficiency SVI

Additional analysis on the effects of tropical cyclone exposure on the SVI subgroups revealed an overall upward trend of the SVI subgroup of household characteristics. To explore plausible underlying mechanisms, we further conducted the outcome analysis on individual variables within this subgroup, with the results shown in Fig. S21. In particular, the effects of tropical cyclones on the percentage of single-parent households were consistently positive, suggesting that tropical cyclones may had led to a profound and lasting change in household structures, possibly driven by factors such as parental mortality, forced displacement, economic strain on families, or lack of social support and policy intervention. On the other hand, other demographic factors contributing to the SVI, such as the percentage of older adults (age 65 years and above), had decreased; while the percentage of young people (under age 17 years) had increased. These shifts in the population age structure suggest that post-disaster development projects may had attracted more young, possibly affluent, young families; while older residents may have left the affected areas. Finally, the percentage of the disabled population initially decreased below the baseline one year after the tropical cyclone, but consistently rebounded over time, surpassing the baseline by 11 years post-exposure.

Socially vulnerable populations–such as single parents, the elderly, children, and individuals with disabilities–are more at greater risk than other groups due to limited resources [11, 12]. The factors driving migration in the aftermath of tropical cyclones are complex. In summary, the observed patterns may reflect broader migration patterns of populations after disasters and underscore the intricate relationship between tropical cyclones and social vulnerability. They also highlight the need for comprehensive health support and disaster relief programs to address the delayed effects of such events. However, further investigation is warranted to validate the hypotheses presented.

4 Sensitivity Analyses

We conducted several sensitivity analyses to assess the robustness of our results to different design and data choices. To address the issue of moderate covariate imbalance for a few exposure years, we performed an outcome analysis on pooled estimates, excluding all $\text{ACEE}_{T_0,\tau}$ estimates where

the mean ASMD across covariates for exposure years was ≥ 0.1. In addition, to verify that the observed downward effect of tropical cyclone exposures on SVI was consistent even without any overlap between exposure and outcome data sources, we conducted an outcome analysis strictly excluding years where the collection periods of tropical cyclone exposure data and SVI data from American Community Survey (ACS) data had overlapped.

4.1 Outcome Analysis Excluding Poorly Balanced Exposure Years

To assess whether the imbalances of synthetic controls introduced bias in our analysis, we applied a mean ASMD threshold of < 0.1 and conducted the same weighted outcome analysis on the pooled ACEE estimates, excluding the poorly balanced exposure years (i.e., 2009, 2014, and 2016). As detailed in Tables S5, S10 and Figs. S8, S13, the exposed units in these years are characterized by a combination of high prior tropical cyclone exposure and high vulnerability, which may be because they fall outside the convex hull of the donor pool [2]. While this structural limitation could potentially be addressed in future work by fitting an outcome model (e.g., as in the Augmented Synthetic Control Method) to correct for interpolation bias [9], here we adopt a conservative approach by excluding these outlier cohorts. The results, shown in Fig. S22, revealed that overall downward trend remained consistent with the main analysis; while the confidence interval (CI) inflated slightly, particularly on the left side of the curve, compared to the main analysis results.

4.2 Outcome Analysis with Strictly Non-overlap Between Exposure and Outcome Data Sources

SVI data were sourced from 5-year ACS data beginning in 2010, which may lead to temporal overlaps between exposure and ACS data sources. Although reverse causation is not of concern in this case–since it is implausible that community social vulnerability would have a causal effect on natural disasters such as tropical cyclones–temporal confounding or post-exposure bias could still complicate the interpretation of the speific impact of tropical cyclones on SVI. To address this, we excluded $\text{ACEE}_{T_0,\tau}$ estimates where there was overlap between the exposure years and the outcome period. For example, for the 2010 SVI, which was constructed based on 5-year ACS data from 2006 to 2010, we only considered the effects of the 2005 exposure on this outcome. Note that SVI in 2010 had to be excluded from the outcome model, as a single observation (i.e., tropical cyclone exposure from 2005) was insufficient to construct an outcome regression model. The results, displayed in Fig. S23, indicate that the long-term dipping effect of tropical cyclones on SVI was persistent, though the jackknife CI's became much wider due to reduced power. Overall, the sensitivity analyses confirmed the robustness of our main results.

5 Software

Software in the form of R code to implement all analyses reported in the paper is available at https://github.com/LincoleJ/tropical_cyclone_svi.git

References

[1] Gould, K.A., Lewis, T.L.: Resilience gentrification: Environmental privilege in an age of coastal climate disasters. Frontiers in Sustainable Cities **3**, 687670 (2021)

[2] Abadie, A.: Using synthetic controls: Feasibility, data requirements, and methodological aspects. Journal of economic literature **59**(2), 391–425 (2021)

[3] Zhao, Q.: Covariate balancing propensity score by tailored loss functions. The Annals of Statistics (2019)

[4] Parast, L., Hunt, P., Griffin, B.A., Powell, D.: When is a match sufficient? a score-based balance metric for the synthetic control method. Journal of Causal Inference **8**(1), 209–228 (2020)

[5] Wu, X., Sverdrup, E., Mastrandrea, M.D., Wara, M.W., Wager, S.: Low-intensity fires mitigate the risk of high-intensity wildfires in california's forests. Science advances **9**(45), 4123 (2023)

[6] Abadie, A., Diamond, A., Hainmueller, J.: Comparative politics and the synthetic control method. American Journal of Political Science **59**(2), 495–510 (2015)

[7] Botosaru, I., Ferman, B.: On the role of covariates in the synthetic control method. The Econometrics Journal **22**(2), 117–130 (2019)

[8] Abadie, A., Diamond, A., Hainmueller, J.: Synthetic control methods for comparative case studies: Estimating the effect of california's tobacco control program. Journal of the American statistical Association **105**(490), 493–505 (2010)

[9] Ben-Michael, E., Feller, A., Rothstein, J.: The augmented synthetic control method. Journal of the American Statistical Association **116**(536), 1789–1803 (2021)

[10] Beven-II, J.L., Avila, L.A., Blake, E.S., Brown, D.P., Franklin, J.L., Knabb, R.D., Pasch, R.J., Rhome, J.R., Stewart, S.R.: Annual summary-atlantic hurricane season of 2005. Tropical Prediction Center, NOAAN/NWS/National Hurricane Center, Miami (March 2008) (2008)

[11] Sastry, N., Gregory, J.: The effect of hurricane katrina on the prevalence of health impairments and disability among adults in new orleans: differences by age, race, and sex. Social Science & Medicine **80**, 121–129 (2013)

[12] Lee, J.Y., Van Zandt, S.: Housing tenure and social vulnerability to disasters: A review of the evidence. Journal of planning literature **34**(2), 156–170 (2019)

Table S1: Data Sources

	Data Source	Github Repository
Outcome & Exposure		
Social Vulnerability Index	CDC/ATSDR	
Tropical Cyclone Exposure	HURDAT	hurricaneexposure, hurricaneexposuredata
Demographic Covariates		
Percentage Black Population	CDC Wonder	cdc_population_monthly_infer
Percentage Hispanic Population	CDC Wonder	cdc_population_monthly_infer
Male / Female Ratio	CDC Wonder	cdc_population_monthly_infer
Population Size	CDC Wonder	cdc_population_monthly_infer
Per Capita Income	BEA	covariates_annual_infer
Percentage Below Poverty Threshold	USCB	covariates_annual_infer
Percentage High School Graduate	USDA	covariates_annual_infer
Meteorological Covariates		
Summer Temperature mean	PRISM	PRISM-grids-into-FIPS-ZIP-censustract-USA
Summer Precipitation mean	PRISM	PRISM-grids-into-FIPS-ZIP-censustract-USA

Table S2: Covariate Means Prior to 2006 Tropical Cyclone Exposure

Covariates	Exposed Regions	Unexposed Regions	Synthetic Control Regions
Past Tropical Cyclone Exposure	0.37	0.04	0.35
Past SVI	0.58	0.50	0.61
Summer Temperature Mean	25.2	23.1	25.1
Summer Precipitation Mean	4.2	3.0	4.2
% High School Grad	74.9	77.1	74.9
% Black	31.9	8.3	31.3
% Hispanic	3.1	6.2	3.1
% Below Poverty	14.7	14.3	14.8
Per Capita Income	25,345	23,634	25,234
Male / Female Ratio	1:1.03	1:1.02	1:1.03
% Youth	20.0	20.7	20.0
% Elderly	14.3	15.0	14.3
Population Density	491	236	490
Population Size	96,600	89,920	100,783

Table S3: Covariate Means Prior to 2007 Tropical Cyclone Exposure

Covariates	Exposed Regions	Unexposed Regions	Synthetic Control Regions
Past Tropical Cyclone Exposure	0.19	0.05	0.19
Past SVI	0.68	0.49	0.67
Summer Temperature Mean	27.0	23.1	27.0
Summer Precipitation Mean	3.9	3.0	3.9
% High School Grad	75.9	77.4	75.9
% Black	16.9	8.9	17.0
% Hispanic	6.7	6.2	6.8
% Below Poverty	16.8	14.3	16.8
Per Capita Income	23,015	23,871	23,005
Male / Female Ratio	1:1.00	1:1.02	1:1.00
% Youth	21.3	20.6	21.3
% Elderly	14.0	15.0	14.0
Population Density	133	249	134
Population Size	111,304	89,993	110,995

Table S4: Covariate Means Prior to 2008 Tropical Cyclone Exposure

Covariates	Exposed Regions	Unexposed Regions	Synthetic Control Regions
Past Tropical Cyclone Exposure	0.17	0.03	0.17
Past SVI	0.56	0.49	0.56
Summer Temperature Mean	25.0	22.9	25.0
Summer Precipitation Mean	3.84	2.89	3.84
% High School Grad	76.5	77.9	76.5
% Black	15.9	7.8	15.9
% Hispanic	7.1	6.2	7.1
% Below Poverty	14.6	14.4	14.6
Per Capita Income	25,000	23,871	25,009
Male / Female Ratio	1:1.02	1:1.01	1:1.02
% Youth	20.5	20.5	20.5
% Elderly	14.5	15.1	14.5
Population Density	712	154	709
Population Size	153,491	78,613	153,627

Table S5: Covariate Means Prior to 2009 Tropical Cyclone Exposure

Covariates	Exposed Regions	Unexposed Regions	Synthetic Control Regions
Past Tropical Cyclone Exposure	0.43	0.06	0.37
Past SVI	0.72	0.50	0.64
Summer Temperature Mean	27.1	23.2	26.9
Summer Precipitation Mean	5.42	3.06	5.30
% High School Grad	73.4	78.0	72.7
% Black	11.9	9.2	12.3
% Hispanic	2.4	6.5	3.3
% Below Poverty	17.7	14.5	17.4
Per Capita Income	21,478	24,235	21,604
Male / Female Ratio	1.12:1	1:1.01	1.11:1
% Youth	17.9	20.4	18.2
% Elderly	15.4	15.0	15.5
Population Density	61.2	247	110
Population Size	43,863	91,593	55,384

Table S6: Covariate Means Prior to 2010 Tropical Cyclone Exposure

Covariates	Exposed Regions	Unexposed Regions	Synthetic Control Regions
Past Tropical Cyclone Exposure	0.18	0.05	0.14
Past SVI	0.84	0.50	0.83
Summer Temperature Mean	29.1	23.1	29.0
Summer Precipitation Mean	2.4	3.1	2.5
% High School Grad	68.0	78.3	67.0
% Black	3.2	9.24	4.3
% Hispanic	57.0	6.2	56.7
% Below Poverty	22.1	14.6	21.8
Per Capita Income	21,709	24,389	21,914
Male / Female Ratio	1.04:1	1:1.01	1.03:1
% Youth	22.5	20.4	22.5
% Elderly	13.4	15.1	13.3
Population Density	142	249	116
Population Size	149,400	91,538	148,120

Table S7: Covariate Means Prior to 2011 Tropical Cyclone Exposure

Covariates	Exposed Regions	Unexposed Regions	Synthetic Control Regions
Past Tropical Cyclone Exposure	0.27	0.04	0.26
Past SVI	0.53	0.50	0.52
Summer Temperature Mean	23.7	23.2	23.7
Summer Precipitation Mean	4.0	3.0	4.0
% High School Grad	79.7	78.5	79.6
% Black	20.5	8.5	20.5
% Hispanic	5.1	6.8	5.1
% Below Poverty	13.5	14.9	13.5
Per Capita Income	29,065	24,247	29,084
Male / Female Ratio	1:1.03	1:1.01	1:1.03
% Youth	19.9	20.3	19.8
% Elderly	13.8	15.2	13.7
Population Density	1,528	165	1,508
Population Size	231,038	83,292	231,010

Table S8: Covariate Means Prior to 2012 Tropical Cyclone Exposure

Covariates	Exposed Regions	Unexposed Regions	Synthetic Control Regions
Past Tropical Cyclone Exposure	0.18	0.05	0.18
Past SVI	0.54	0.50	0.55
Summer Temperature Mean	24.5	23.2	24.5
Summer Precipitation Mean	4.0	3.0	3.0
% High School Grad	79.3	78.8	79.3
% Black	18.7	8.6	18.5
% Hispanic	5.3	6.9	5.4
% Below Poverty	13.8	15.0	13.9
Per Capita Income	29,035	24,464	28,937
Male / Female Ratio	1:1.02	1:1.01	1:1.02
% Youth	20.1	20.2	20.1
% Elderly	13.6	15.3	13.6
Population Density	1652	156	1611
Population Size	239,060	83,013	237,506

Table S9: Covariate Means Prior to 2013 Tropical Cyclone Exposure

Covariates	Exposed Regions	Unexposed Regions	Synthetic Control Regions
Past Tropical Cyclone Exposure	0.38	0.04	0.36
Past SVI	0.67	0.50	0.68
Summer Temperature Mean	25.9	23.3	25.8
Summer Precipitation Mean	4.6	3.0	4.5
% High School Grad	77.4	79.2	77.4
% Black	30.3	8.7	29.5
% Hispanic	3.8	7.0	4.1
% Below Poverty	16.8	15.0	16.7
Per Capita Income	25,032	24,965	24,978
Male / Female Ratio	1:1.02	1:1.01	1:1.02
% Youth	20.0	20.2	20.0
% Elderly	13.9	15.3	14.0
Population Density	346	248	338
Population Size	86,197	93,441	91,237

Table S10: Covariate Means Prior to 2014 Tropical Cyclone Exposure

Covariates	Exposed Regions	Unexposed Regions	Synthetic Control Regions
Past Tropical Cyclone Exposure	0.50	0.04	0.28
Past SVI	0.70	0.50	0.68
Summer Temperature Mean	25.6	23.3	25.7
Summer Precipitation Mean	4.5	3.1	4.4
% High School Grad	77.3	79.5	75.3
% Black	34.0	8.9	31.9
% Hispanic	3.8	7.1	4.0
% Below Poverty	17.5	15.1	18.0
Per Capita Income	24,687	25,178	24,163
Male / Female Ratio	1:1.04	1:1.01	1:1.03
% Youth	19.7	20.1	20.1
% Elderly	14.4	15.4	14.5
Population Density	407	249	290
Population Size	77,278	93,965	77,693

Table S11: Covariate Means Prior to 2015 Tropical Cyclone Exposure

Covariates	Exposed Regions	Unexposed Regions	Synthetic Control Regions
Past Tropical Cyclone Exposure	0.27	0.05	0.26
Past SVI	0.80	0.50	0.75
Summer Temperature Mean	28.1	23.2	27.9
Summer Precipitation Mean	3.4	3.1	3.4
% High School Grad	74.6	79.7	74.5
% Black	13.9	9.3	14.0
% Hispanic	29.7	7.00	29.0
% Below Poverty	19.0	15.2	18.7
Per Capita Income	24,451	25,370	24,410
Male / Female Ratio	1.02:1	1:1.01	1.02:1
% Youth	20.4	20.0	20.4
% Elderly	15.5	15.5	15.4
Population Density	114	253	113
Population Size	59,702	94,295	67,721

Table S12: Covariate Means Prior to 2016 Tropical Cyclone Exposure

Covariates	Exposed Regions	Unexposed Regions	Synthetic Control Regions
Past Tropical Cyclone Exposure	0.26	0.04	0.23
Past SVI	0.78	0.48	0.75
Summer Temperature Mean	26.5	23.1	26.5
Summer Precipitation Mean	5.0	3.0	4.8
% High School Grad	75.3	80.2	75.5
% Black	29.3	8.2	28.5
% Hispanic	6.0	7.3	6.5
% Below Poverty	20.0	15.0	19.6
Per Capita Income	22,764	25,724	23,157
Male / Female Ratio	1.01:1	1:1.01	1.01:1
% Youth	19.9	19.9	19.9
% Elderly	14.7	15.7	15.0
Population Density	140	260	225
Population Size	108,493	93,706	117,590

Table S13: Covariate Means Prior to 2017 Tropical Cyclone Exposure

Covariates	Exposed Regions	Unexposed Regions	Synthetic Control Regions
Past Tropical Cyclone Exposure	0.23	0.04	0.23
Past SVI	0.74	0.49	0.68
Summer Temperature Mean	27.8	23.1	27.4
Summer Precipitation Mean	4.8	3.1	4.5
% High School Grad	75.8	80.5	76.2
% Black	17.3	9.0	16.8
% Hispanic	17.6	6.8	16.7
% Below Poverty	18.6	15.2	18.6
Per Capita Income	24,371	25,809	24.191
Male / Female Ratio	1.02:1	1:1.01	1.01:1
% Youth	20.2	19.8	20.1
% Elderly	15.6	15.8	15.5
Population Density	216	256	176
Population Size	160,534	91,470	142,647

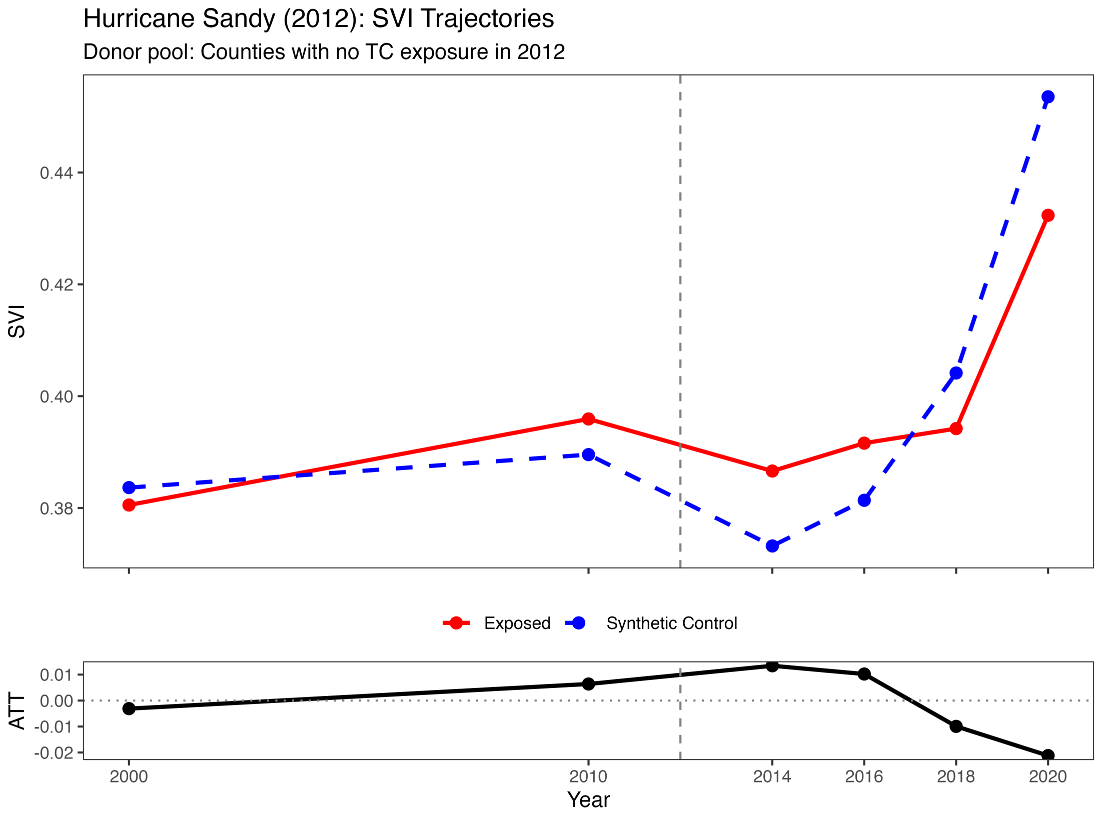

Fig. S1: Evolution of overall SVI for counties exposed to Hurricane Sandy at gale force level (≥ 34 knots) from 2000 to 2020, compared to that of the weighted synthetic control.

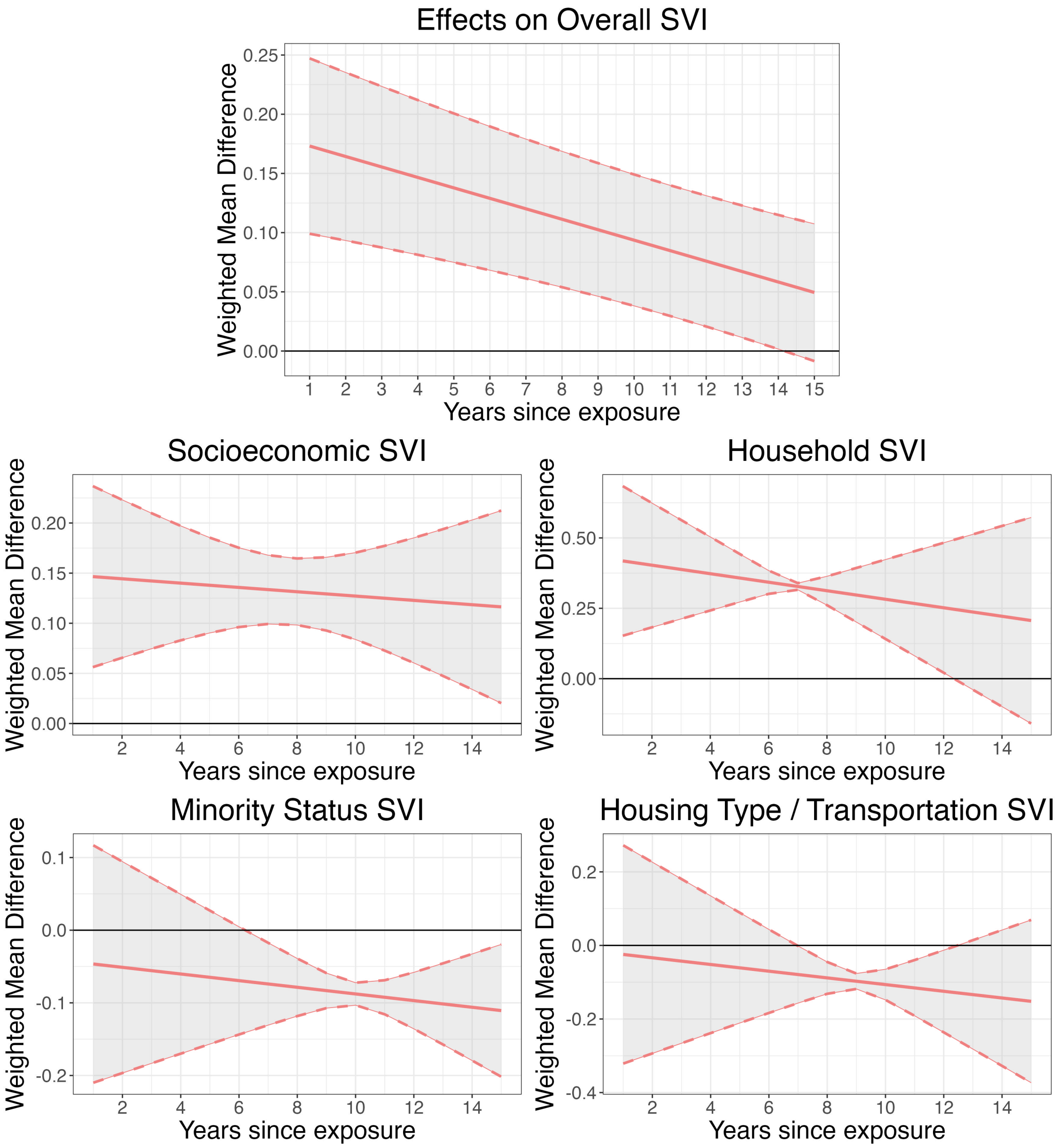

Fig. S2: Effects of hurricane exposures on community social vulnerability up to 15-year lags, pooled across exposure years 2005, 2008, 2017, and 2018. Two-sided 95% CIs are presented.

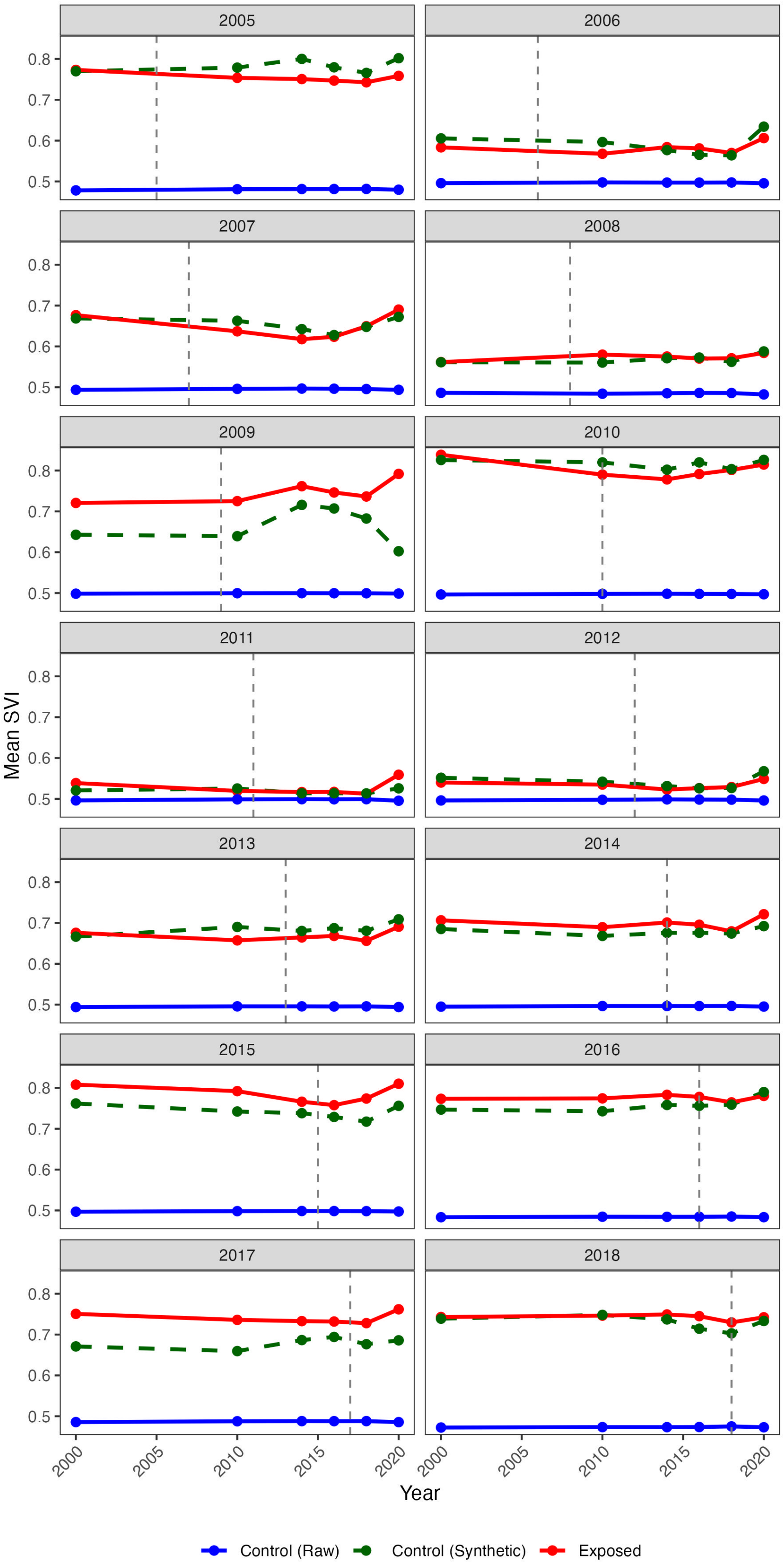

Fig. S3: Raw Social Vulnerability Trends for each exposure year. The panels show the mean SVI values for exposed counties (solid red) compared to the raw average of the unexposed counties (solid blue) and the weighted synthetic controls (dashed green). The vertical dashed gray line indicates the year of tropical cyclone exposure.

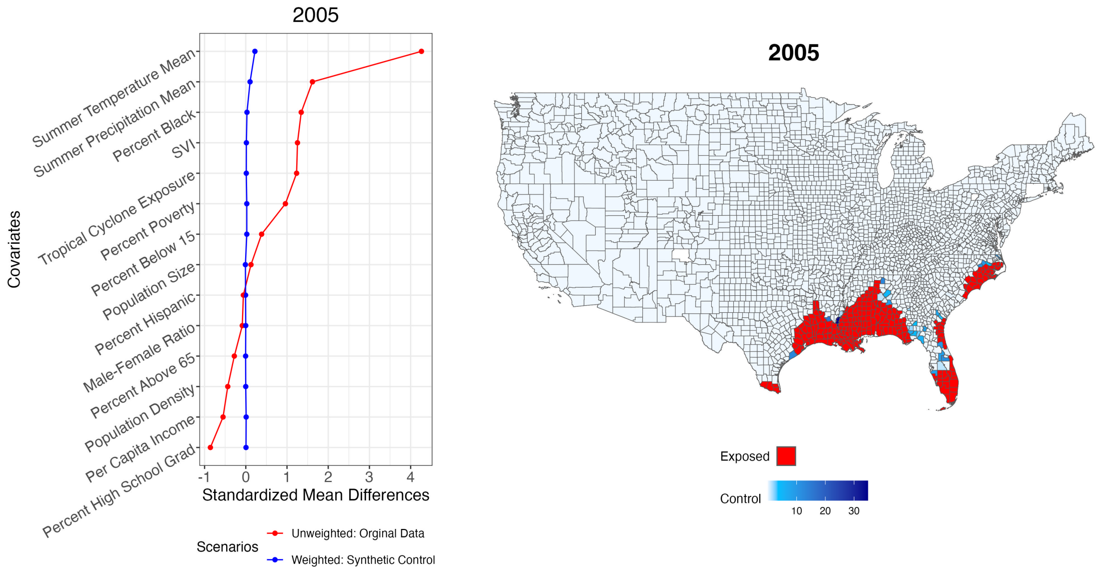

Fig. S4: Covariate balance and exposed/control region distribution under the synthetic control experimental design for the exposure year 2005

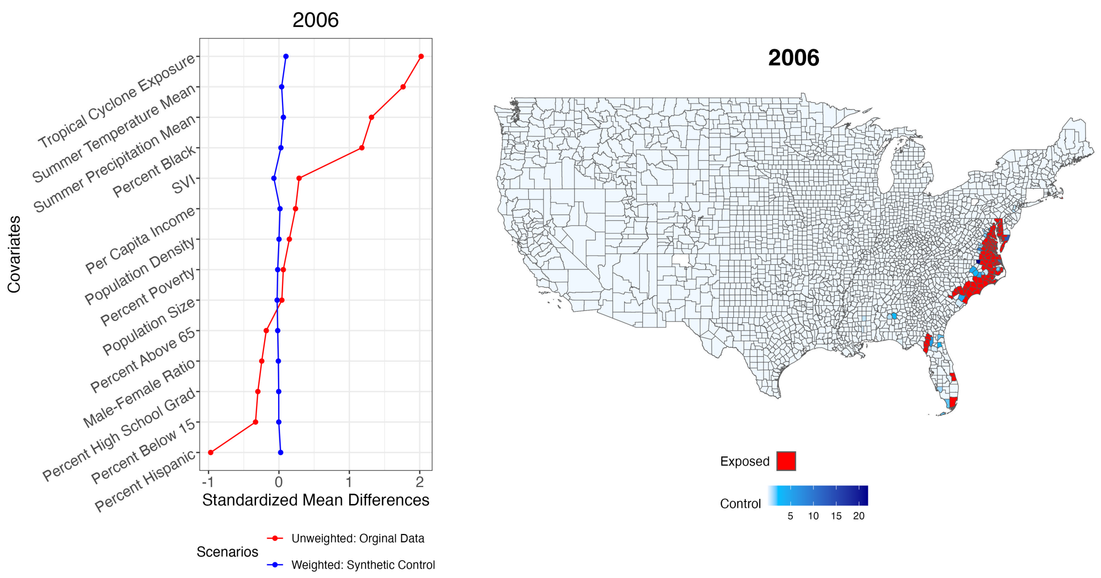

Fig. S5: Covariate balance and exposed/control region distribution under the synthetic control experimental design for the exposure year 2006

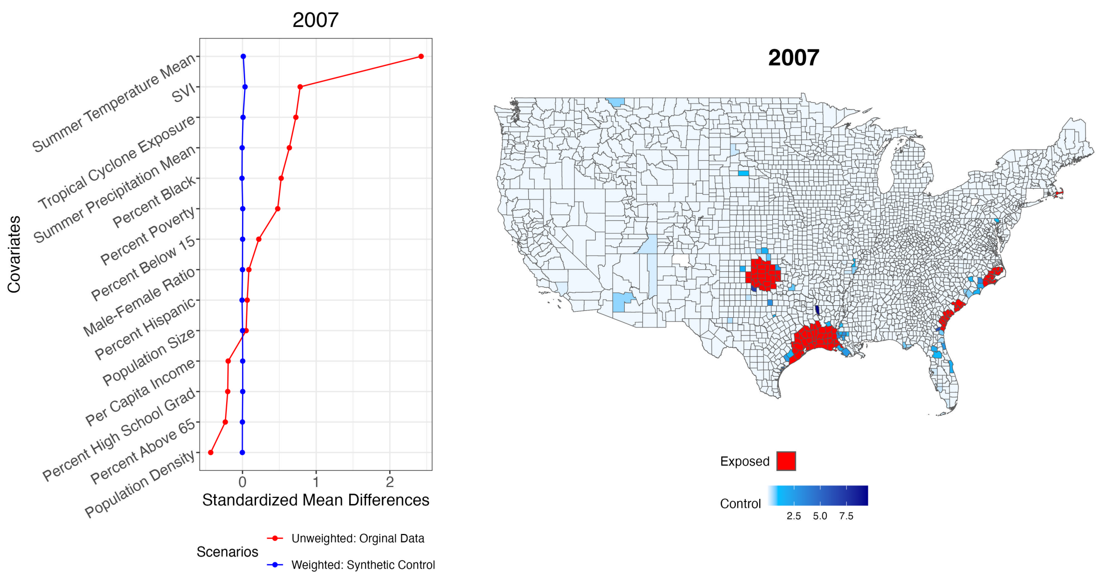

Fig. S6: Covariate balance and exposed/control region distribution under the synthetic control experimental design for the exposure year 2007

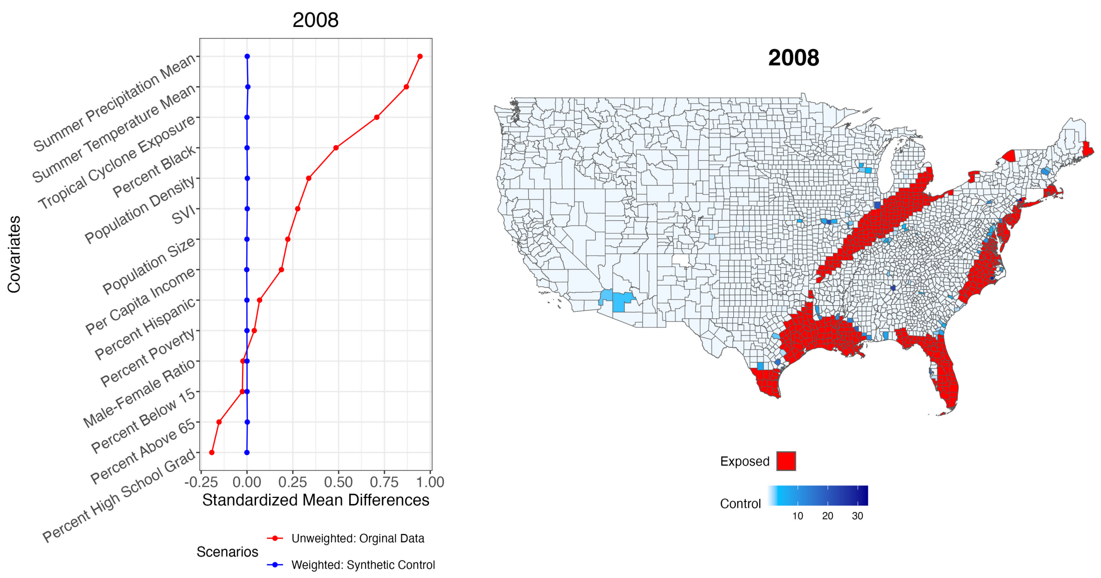

Fig. S7: Covariate balance and exposed/control region distribution under the synthetic control experimental design for the exposure year 2008

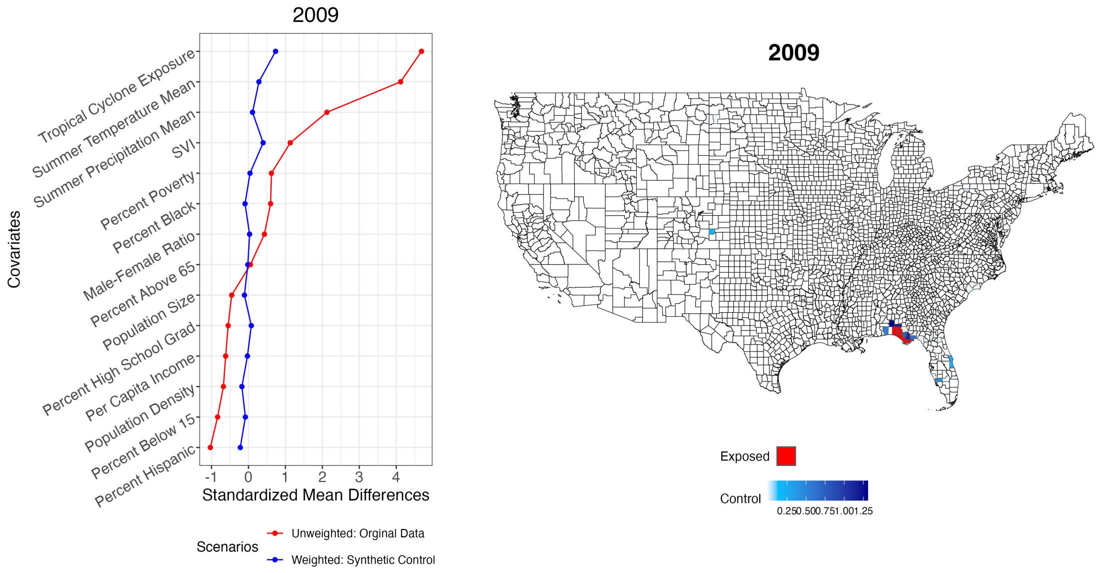

Fig. S8: Covariate balance and exposed/control region distribution under the synthetic control experimental design for the exposure year 2009

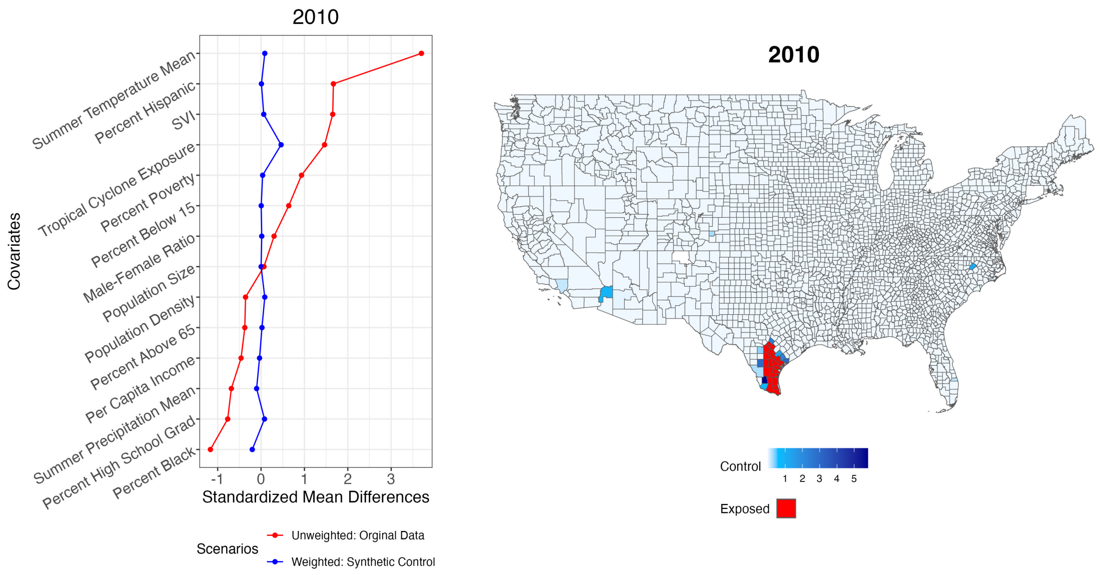

Fig. S9: Covariate balance and exposed/control region distribution under the synthetic control experimental design for the exposure year 2010

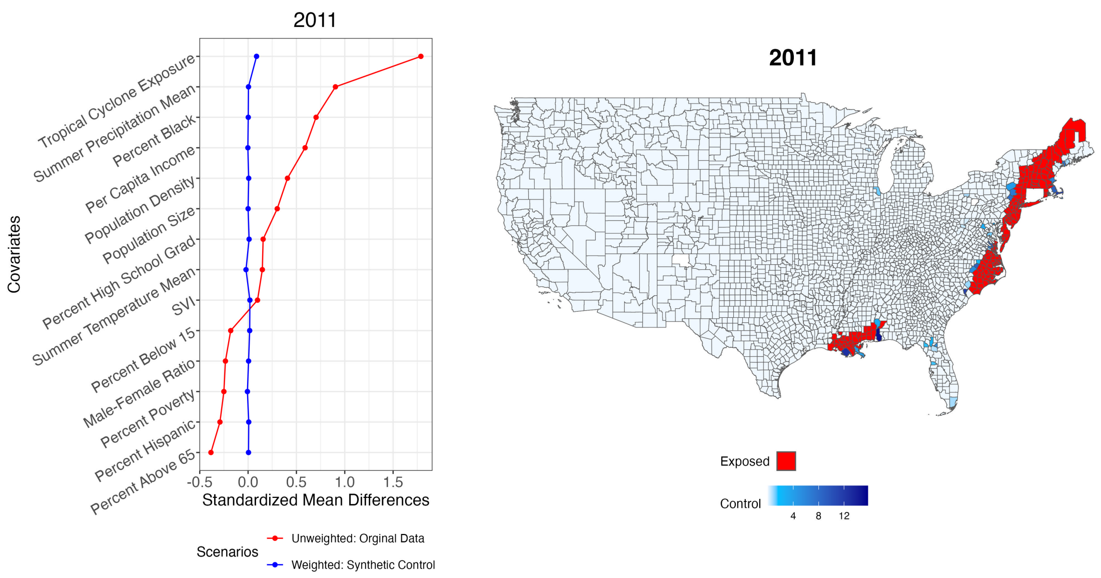

Fig. S10: Covariate balance and exposed/control region distribution under the synthetic control experimental design for the exposure year 2011

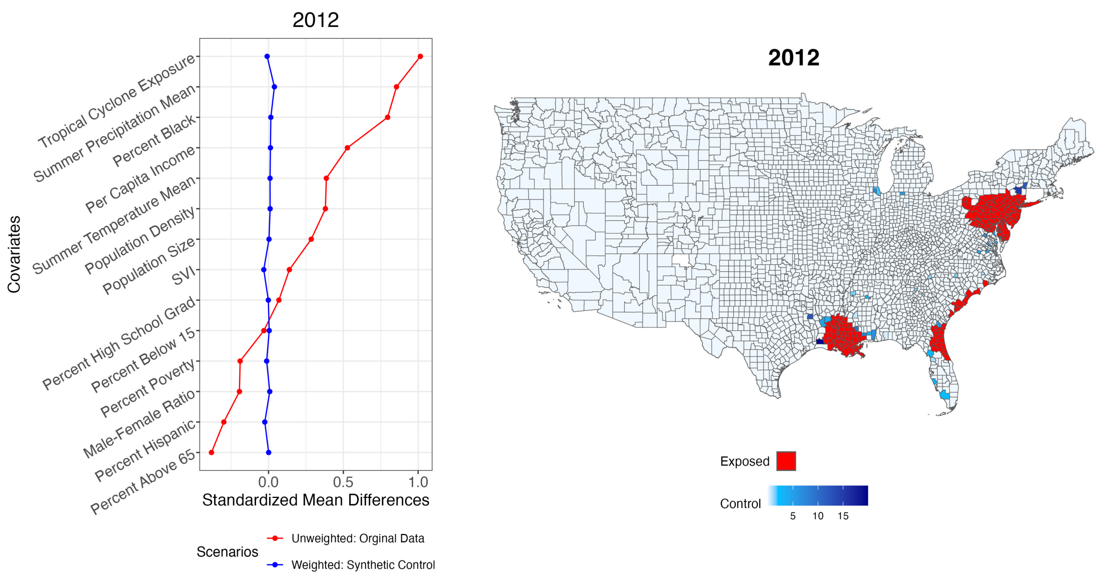

Fig. S11: Covariate balance and exposed/control region distribution under the synthetic control experimental design for the exposure year 2012

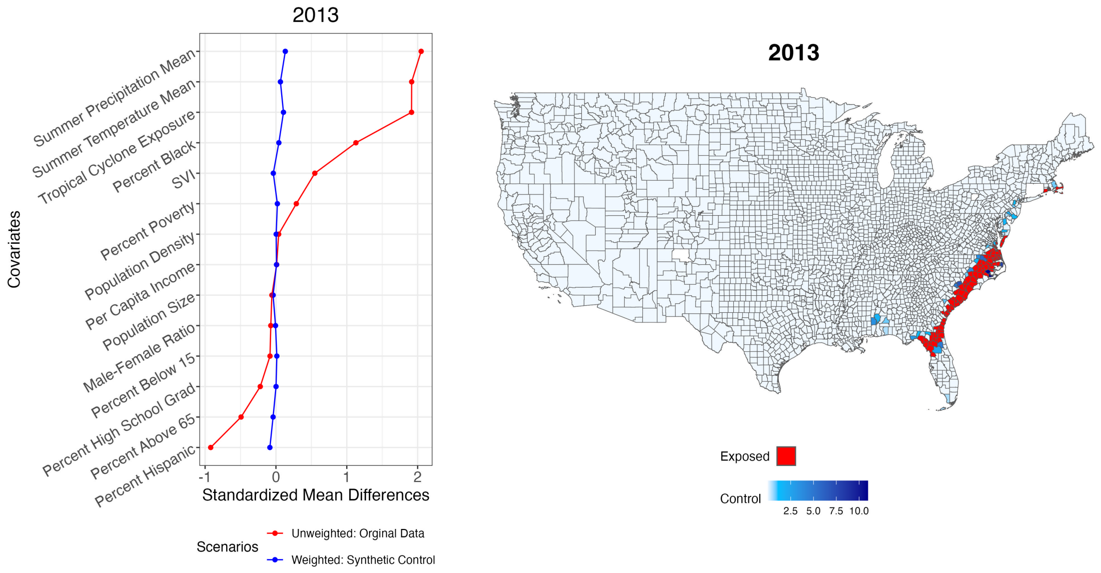

Fig. S12: Covariate balance and exposed/control region distribution under the synthetic control experimental design for the exposure year 2013

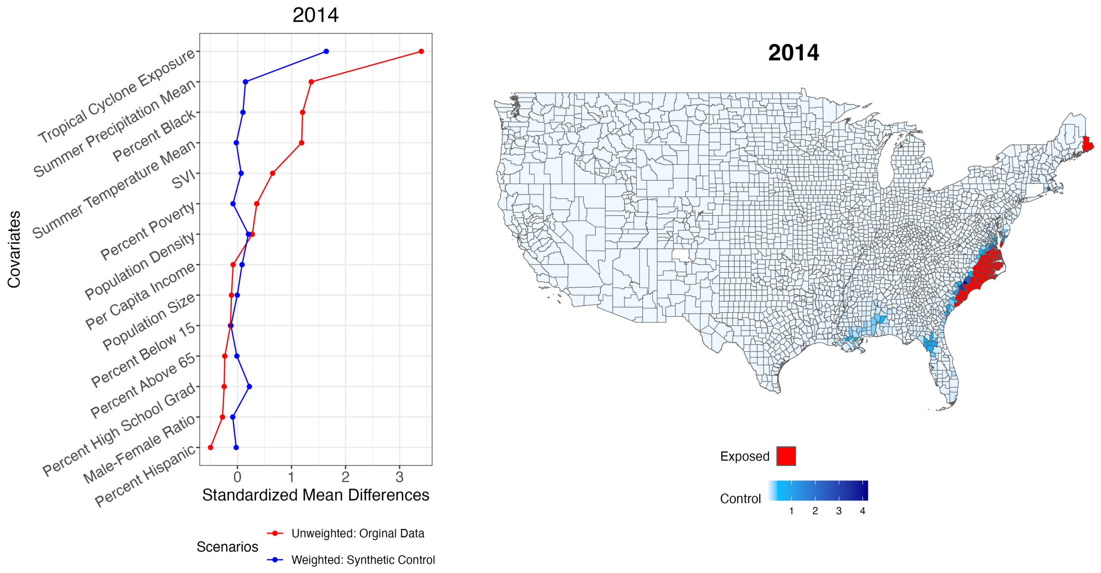

Fig. S13: Covariate balance and exposed/control region distribution under the synthetic control experimental design for the exposure year 2014

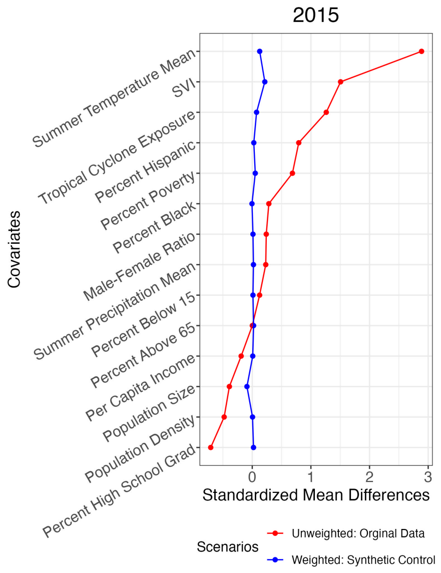

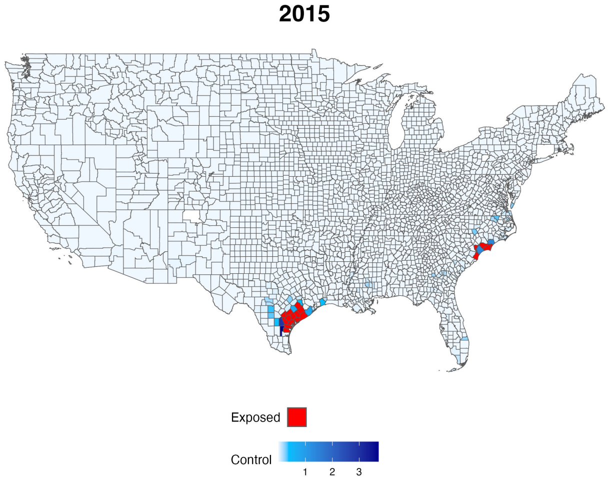

Fig. S14: Covariate balance and exposed/control region distribution under the synthetic control experimental design for the exposure year 2015

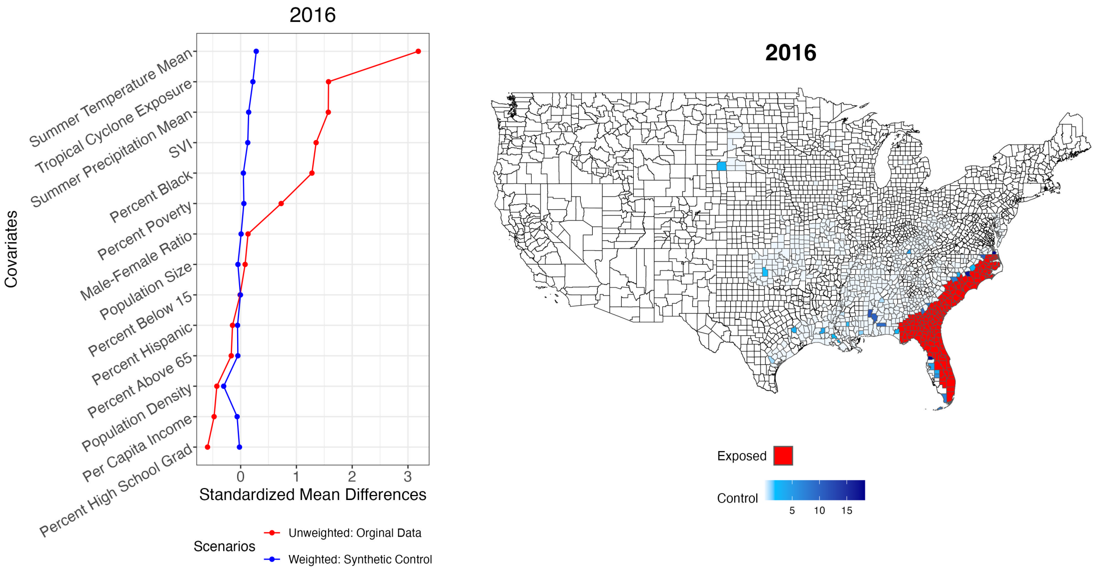

Fig. S15: Covariate balance and exposed/control region distribution under the synthetic control experimental design for the exposure year 2016

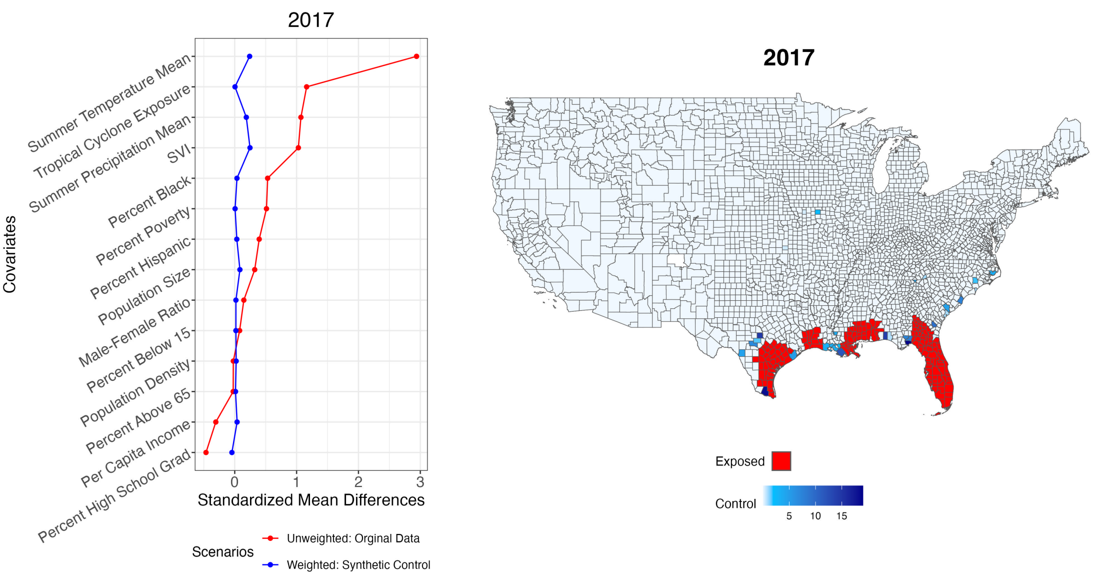

Fig. S16: Covariate balance and exposed/control region distribution under the synthetic control experimental design for the exposure year 2017

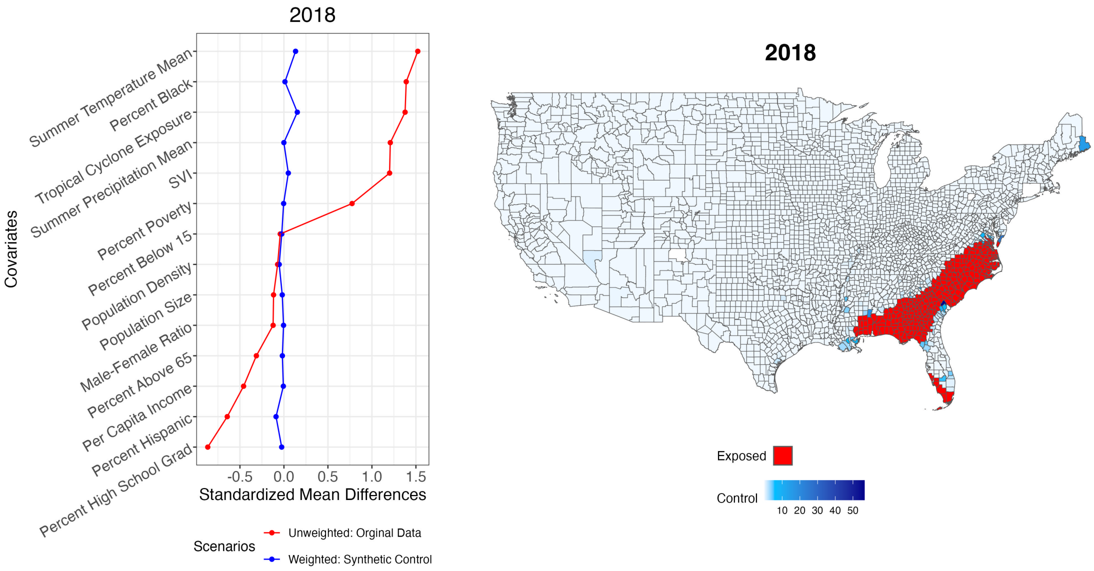

Fig. S17: Covariate balance and exposed/control region distribution under the synthetic control experimental design for the exposure year 2018

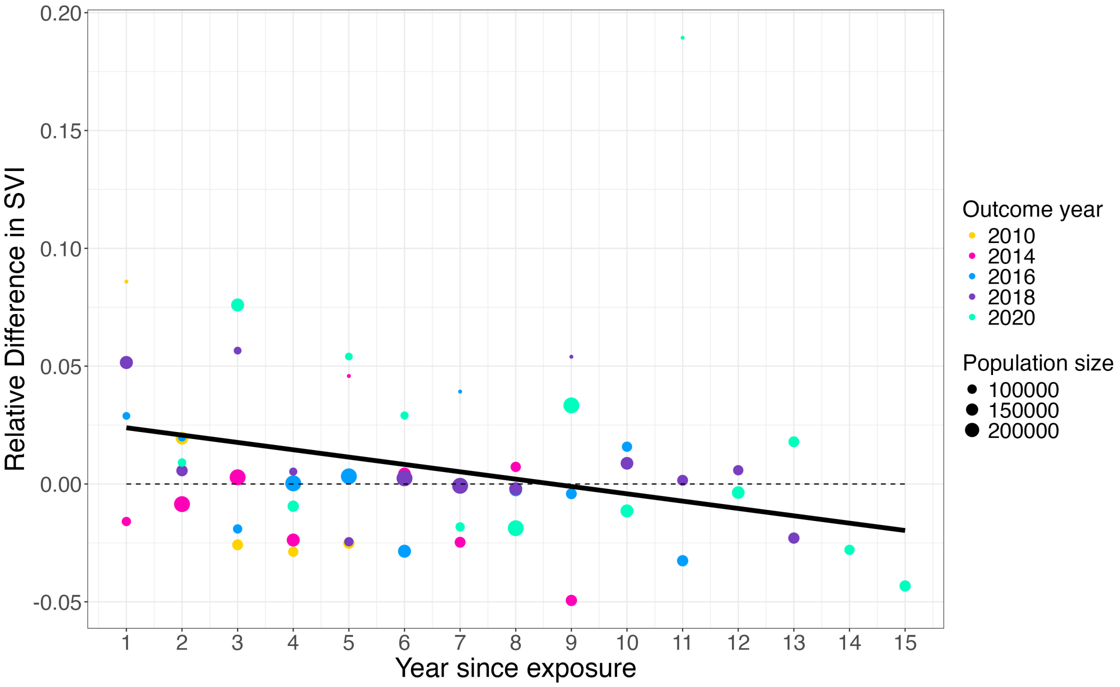

Fig. S18: The individual points display ACEE_{τ,T_0} estimates obtained via synthetic control approaches for each lag τ and outcome year t across all exposure years. The point size measures the population size and quantifies the relative weights of each ACEE estimate (i.e., larger points represent exposed regions with larger population sizes). The thick line corresponds to the ACEE estimate reported in the main text, and is obtained from the individual point estimates via precision-weighted natural splines modeling.

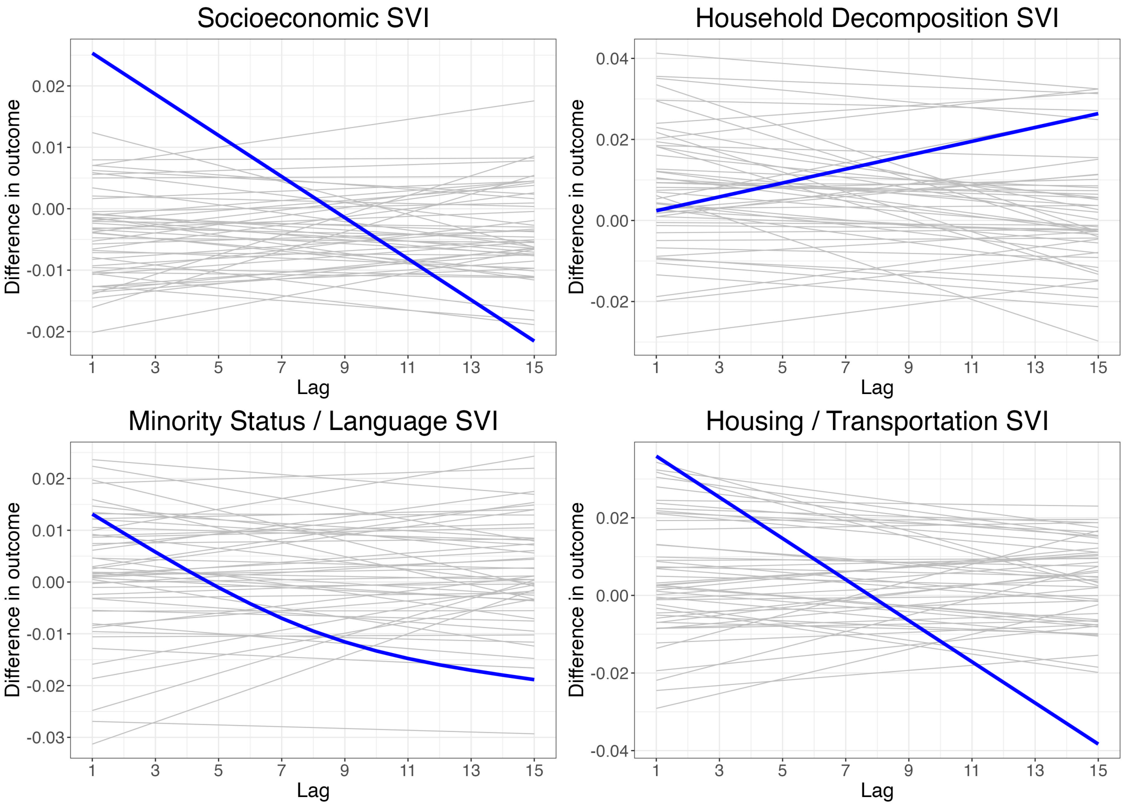

Fig. S19: Placebo test results for domains of SVI. The blue line indicates the outcome model for the actual data; the gray lines indicate each of the 50 placebo runs, respectively

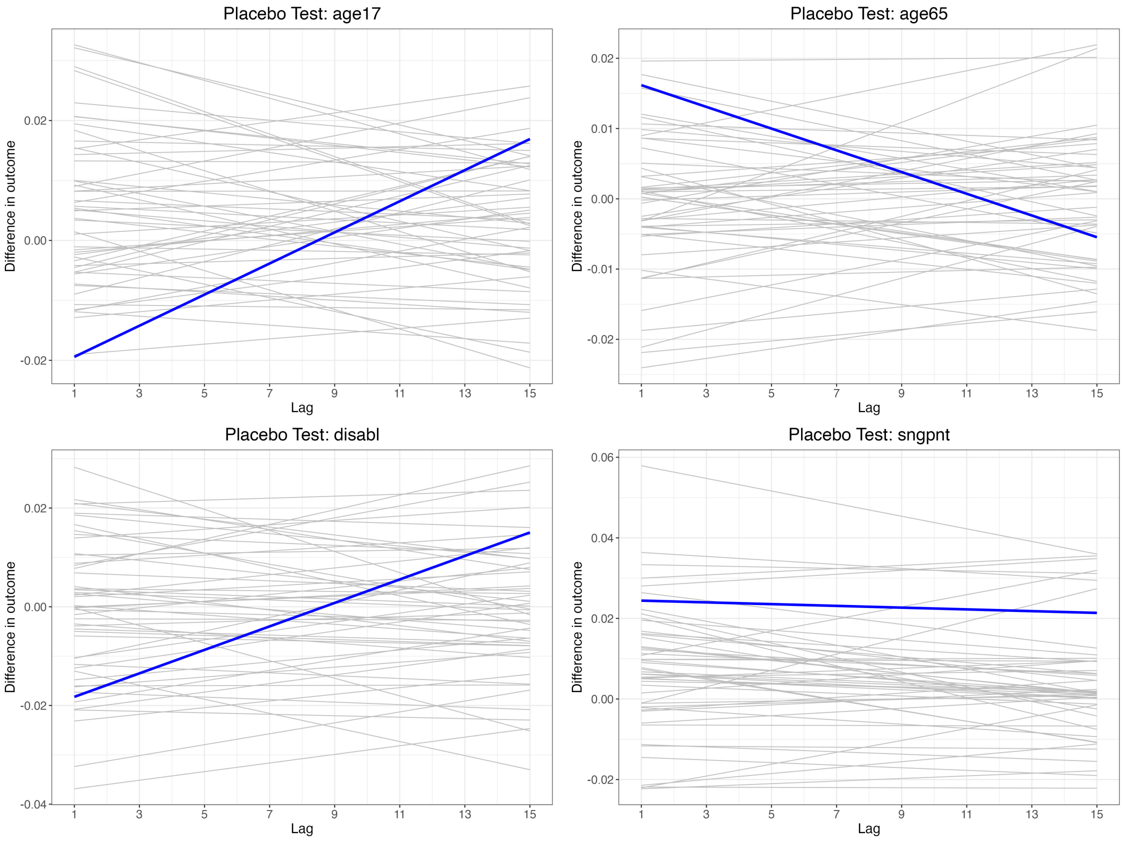

Fig. S20: Placebo test results for variables under the Household Characteristics SVI. The blue line indicates the outcome model for the actual data; the gray lines indicate each of the 50 placebo runs, respectively

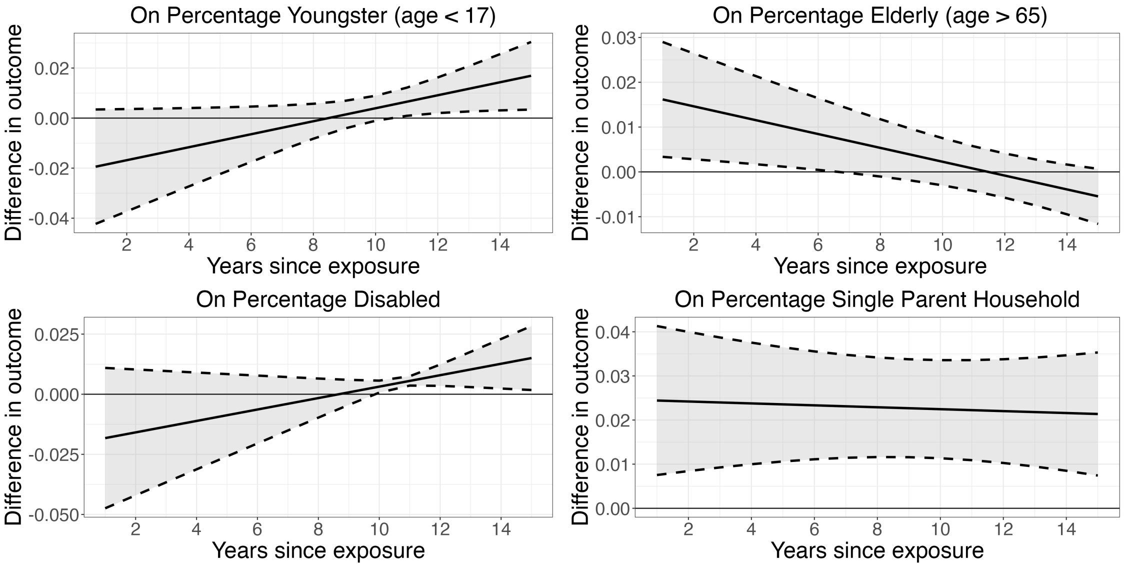

Fig. S21: Outcome analysis results on individual demographic variables under the SVI domain of household characteristics.

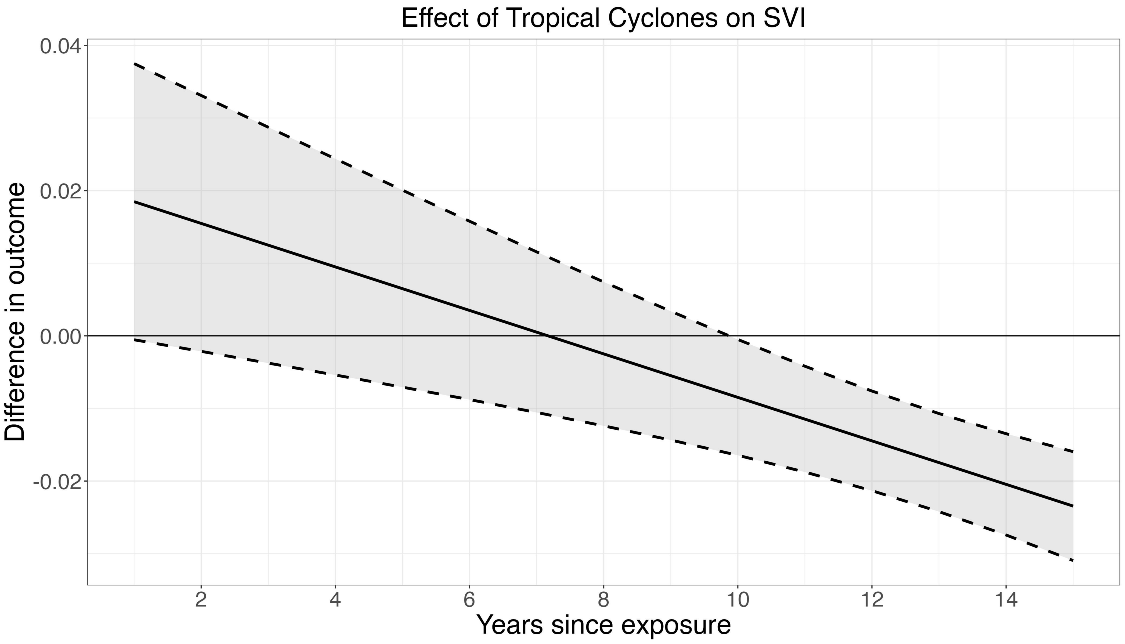

Fig. S22: Outcome analysis results with covariate imbalanced exposure years 2009, 2014, and 2018 excluded. The overall trend is similar despite diminished power due to decreased sample size.

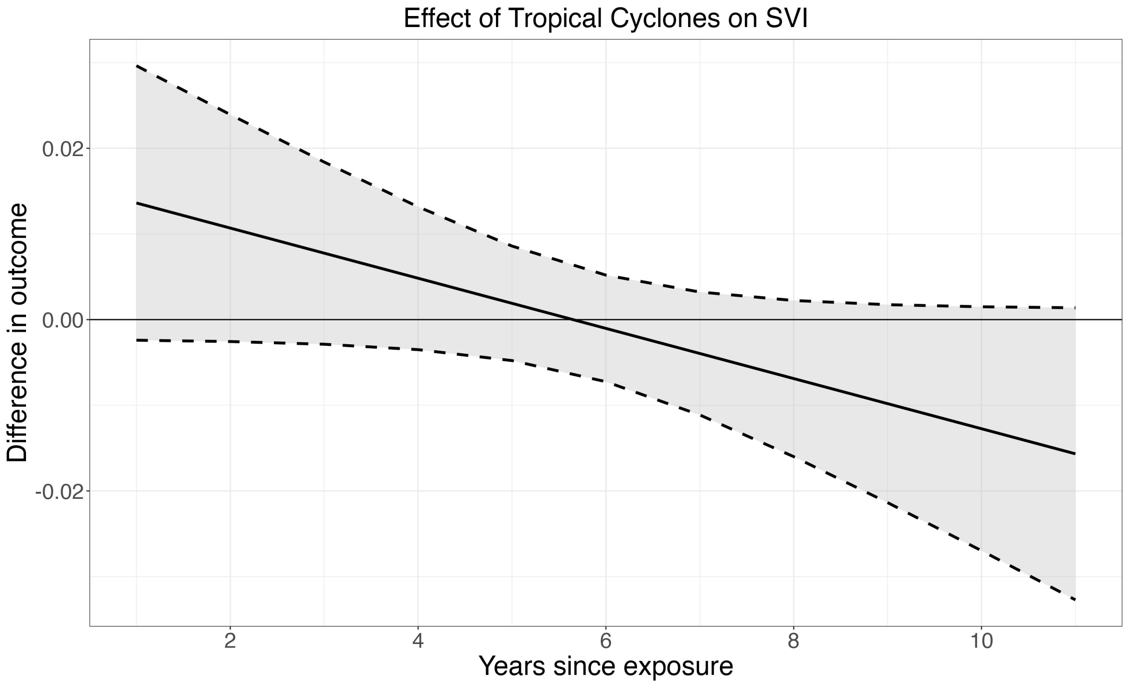

Fig. S23: Outcome analysis results with strictly non-overlap between tropical cyclone exposure and ACS data sources. The overall trend is similar despite diminished power due to the smaller number of pooled estimates.
